# Dopaminergic Neuronal Loss, Reduced Neurite Complexity and Autophagic Abnormalities in Transgenic Mice Expressing G2019S Mutant LRRK2

**DOI:** 10.1371/journal.pone.0018568

**Published:** 2011-04-06

**Authors:** David Ramonet, João Paulo L. Daher, Brian M. Lin, Klodjan Stafa, Jaekwang Kim, Rebecca Banerjee, Marie Westerlund, Olga Pletnikova, Liliane Glauser, Lichuan Yang, Ying Liu, Deborah A. Swing, M. Flint Beal, Juan C. Troncoso, J. Michael McCaffery, Nancy A. Jenkins, Neal G. Copeland, Dagmar Galter, Bobby Thomas, Michael K. Lee, Ted M. Dawson, Valina L. Dawson, Darren J. Moore

**Affiliations:** 1 Brain Mind Institute, School of Life Sciences, Ecole Polytechnique Fédérale de Lausanne (EPFL), Lausanne, Switzerland; 2 NeuroRegeneration and Stem Cell Programs, Institute for Cell Engineering, Johns Hopkins University School of Medicine, Baltimore, Maryland, United States of America; 3 Department of Neurology, Johns Hopkins University School of Medicine, Baltimore, Maryland, United States of America; 4 Department of Pathology, School of Medicine, Fluminense Federal University, Niterói, Brazil; 5 Department of Pathology, Johns Hopkins University School of Medicine, Baltimore, Maryland, United States of America; 6 Institute for Translational Neuroscience, Department of Neuroscience, University of Minnesota, Minneapolis, Minnesota, United States of America; 7 Department of Neurology and Neuroscience, Weill Medical College of Cornell University, New York, New York, United States of America; 8 Department of Neuroscience, Karolinska Institutet, Stockholm, Sweden; 9 Integrated Imaging Center and Department of Biology, Johns Hopkins University, Baltimore, Maryland, United States of America; 10 Mouse Cancer Genetics Program, NCI-Frederick Cancer Research and Development Center, Frederick, Maryland, United States of America; 11 Department of Neuroscience, Johns Hopkins University School of Medicine, Baltimore, Maryland, United States of America; 12 Department of Physiology, Johns Hopkins University School of Medicine, Baltimore, Maryland, United States of America; National Institute of Health, United States of America

## Abstract

Mutations in the *leucine-rich repeat kinase 2* (*LRRK2*) gene cause late-onset, autosomal dominant familial Parkinson's disease (PD) and also contribute to idiopathic PD. *LRRK2* mutations represent the most common cause of PD with clinical and neurochemical features that are largely indistinguishable from idiopathic disease. Currently, transgenic mice expressing wild-type or disease-causing mutants of LRRK2 have failed to produce overt neurodegeneration, although abnormalities in nigrostriatal dopaminergic neurotransmission have been observed. Here, we describe the development and characterization of transgenic mice expressing human LRRK2 bearing the familial PD mutations, R1441C and G2019S. Our study demonstrates that expression of G2019S mutant LRRK2 induces the degeneration of nigrostriatal pathway dopaminergic neurons in an age-dependent manner. In addition, we observe autophagic and mitochondrial abnormalities in the brains of aged G2019S LRRK2 mice and markedly reduced neurite complexity of cultured dopaminergic neurons. These new LRRK2 transgenic mice will provide important tools for understanding the mechanism(s) through which familial mutations precipitate neuronal degeneration and PD.

## Introduction

Mutations in the *LRRK2* gene (PARK8, OMIM 609007) cause late-onset, autosomal dominant familial Parkinson's disease (PD) with a clinical and neurochemical phenotype that is largely indistinguishable from sporadic PD [1]–[3]. At least six disease-segregating mutations have been identified in *LRRK2*-linked families, including the R1441C/G/H, Y1699C, G2019S and I2020T variants [4]–[5]. Of these, G2019S is the most common variant that uniquely contributes to both familial and sporadic PD [6]–[9]. *LRRK2*-linked PD is characterized by the degeneration of substantia nigra dopaminergic neurons and gliosis together with heterogeneous protein inclusion pathology [3], [10]. How mutations in *LRRK2* precipitate neuronal degeneration and pathology in PD is not known.

*LRRK2* encodes a multi-domain protein belonging to the ROCO family characterized by a Ras of Complex (ROC) GTPase domain and a C-terminal of ROC (COR) domain in conjunction with a kinase domain with similarity to RIP kinases [11]–[12]. LRRK2 contains both GTPase and kinase activities and certain familial mutations can modify one or other of these enzymatic activities [5], [11], [13]–[19]. Familial mutations consistently enhance LRRK2-induced neuronal toxicity *in vitro* in a GTP-binding- and kinase-dependent manner [13], [19]-[22], suggesting a gain-of-function mechanism for familial mutations. Whether LRRK2 mutations can also induce neuronal toxicity *in vivo* has not been demonstrated. LRRK2 expression has been shown to regulate neuronal morphology *in vitro* where familial LRRK2 mutants induce a reduction of neurite length and branching, and LRRK2 deficiency produces opposing effects [20]. Autophagy may mediate neurite shortening induced by G2019S LRRK2 expression since inhibition of autophagy reverses, and activation potentiates, the effects of G2019S LRRK2 on neurites [23]. These observations suggest a potential role for autophagy in mediating the pathogenic actions of LRRK2 mutations.

A number of models have been developed to probe the normal function of LRRK2 *in vivo*, and to dissect the pathogenic actions of familial mutations. Genetic disruption of *LRRK2* or its paralogs in *Caenorhabditis elegans* [24]–[25], *Drosophila melanogaster* [26] and mice [27]–[28] suggest that LRRK2 is not essential for the survival of dopaminergic neurons. However, transgenic expression of human LRRK2 bearing the G2019S mutation in *Drosophila* causes adult-onset, selective degeneration of dopaminergic neurons, L-DOPA-responsive locomotor impairment and early mortality [29]–[30]. LRRK2 transgenic mice have been developed recently to model *LRRK2*-linked PD [31]–[35]. BAC transgenic mice expressing R1441G mutant LRRK2 exhibit reduced striatal dopamine release, L-DOPA-sensitive motor deficits, dopaminergic neuritic atrophy/dystrophy and increased tau phosphorylation [32]. Additionally, BAC mice expressing G2019S mutant LRRK2 or R1441C knock-in mice display impairments of nigrostriatal dopaminergic neurotransmission and tau processing [31], [34]–[35]. These mouse models have provided important insight into the pathogenic effects of familial *LRRK2* mutations *in vivo* and further support a gain-of-function mechanism for these mutations. However, the current mouse models do not exhibit overt neuronal loss and have failed to recapitulate the progressive degeneration of nigrostriatal dopaminergic neurons; the hallmark pathology underlying the clinical motor symptoms of PD.

To model the effects of familial mutations *in vivo*, we have developed LRRK2 transgenic mice bearing the PD-associated R1441C and G2019S mutations or wild-type LRRK2. Here, we demonstrate that the expression of G2019S LRRK2 induces the progressive degeneration of nigrostriatal dopaminergic neurons in mice. G2019S LRRK2 expression also produces autophagic and mitochondrial abnormalities in the mouse brain, and reduces dopaminergic neurite complexity in primary cultures. Our study provides new insight into the pathogenic actions of familial LRRK2 mutations *in vivo* related to the pathogenesis of PD, and provides a novel model of dopaminergic neurodegeneration induced by the expression of G2019S mutant LRRK2.

## Results

### Generation of Transgenic Mice Expressing Mutant Human LRRK2

**Figure 1:**
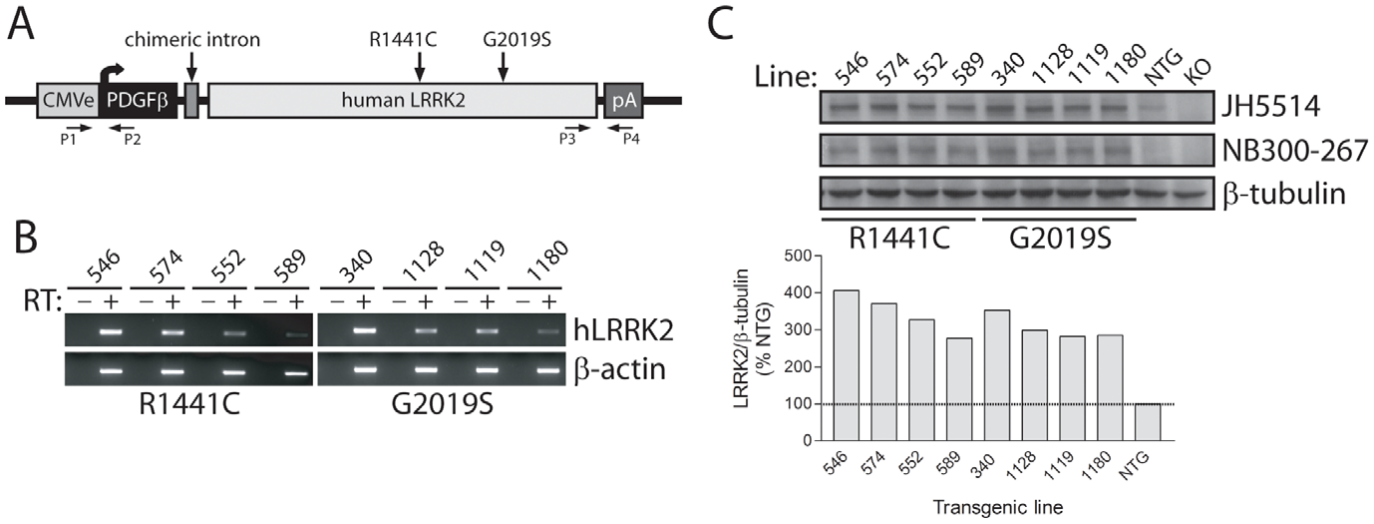
Generation of LRRK2 transgenic mice. ***A***, Schematic showing the CMVE-PDGFβ-LRRK2 transgene and the positions of familial PD mutations. PCR primers for 5′ (P1/P2) and 3′ (P3/P4) genotyping are indicated. ***B***, Semi-quantitative RT-PCR analysis of human LRRK2 mRNA expression in 2–3 month LRRK2 transgenic lines. Mouse β-actin mRNA is used as a loading control. The absence (−) or presence (+) of RT enzyme in the reaction is indicated. ***C***, Western blot analysis of soluble extracts from hemi-brains of 2–3 month LRRK2 transgenic mice (TG), non-transgenic mice (NTG) or LRRK2 knockout (KO) mice using LRRK2-specific antibodies, JH5514 (human/mouse) or human-specific NB300-267. β-tubulin is used a control for protein loading. Bar chart showing densitometric quantitation of total LRRK2 levels (JH5514 antibody) in each transgenic line. LRRK2 levels are normalized to β-tubulin levels and expressed as a percent of NTG mice.

The expression of full-length human LRRK2 variants was placed under the control of a CMV-enhanced human platelet-derived growth factor β-chain (CMVE-PDGFβ) promoter (Figure 1A). This hybrid promoter drives long-term neuronal-specific transgene expression in the rat brain including substantia nigra dopaminergic neurons [36]–[38]. Transgenic mice were generated expressing human LRRK2 harboring the familial PD mutations, R1441C and G2019S, in addition to WT LRRK2. We identified 73 founder mice by genomic PCR with 5′ and 3′ primer pairs (Figure 1A). Quantitative PCR using genomic DNA revealed the relative transgene copy number between founder mice (data not shown). Of the initial founders, 24 lines with medium-high transgene copy number transmitted the transgene to F1 progeny following breeding to C57BL/6J mice. Semi-quantitative RT-PCR revealed the expression levels of human LRRK2 mRNA in hemi-brains of F1 mice (Figure 1B). We selected 4 lines for each LRRK2 variant with the highest transgene expression and determined human LRRK2 protein levels in hemi-brain extracts by Western blotting with pan- or human-specific LRRK2 antibodies. LRRK2 transgenic mice express human LRRK2 at 3-5-fold the level of endogenous LRRK2 (Figure 1C and S1).

**Figure 2:**
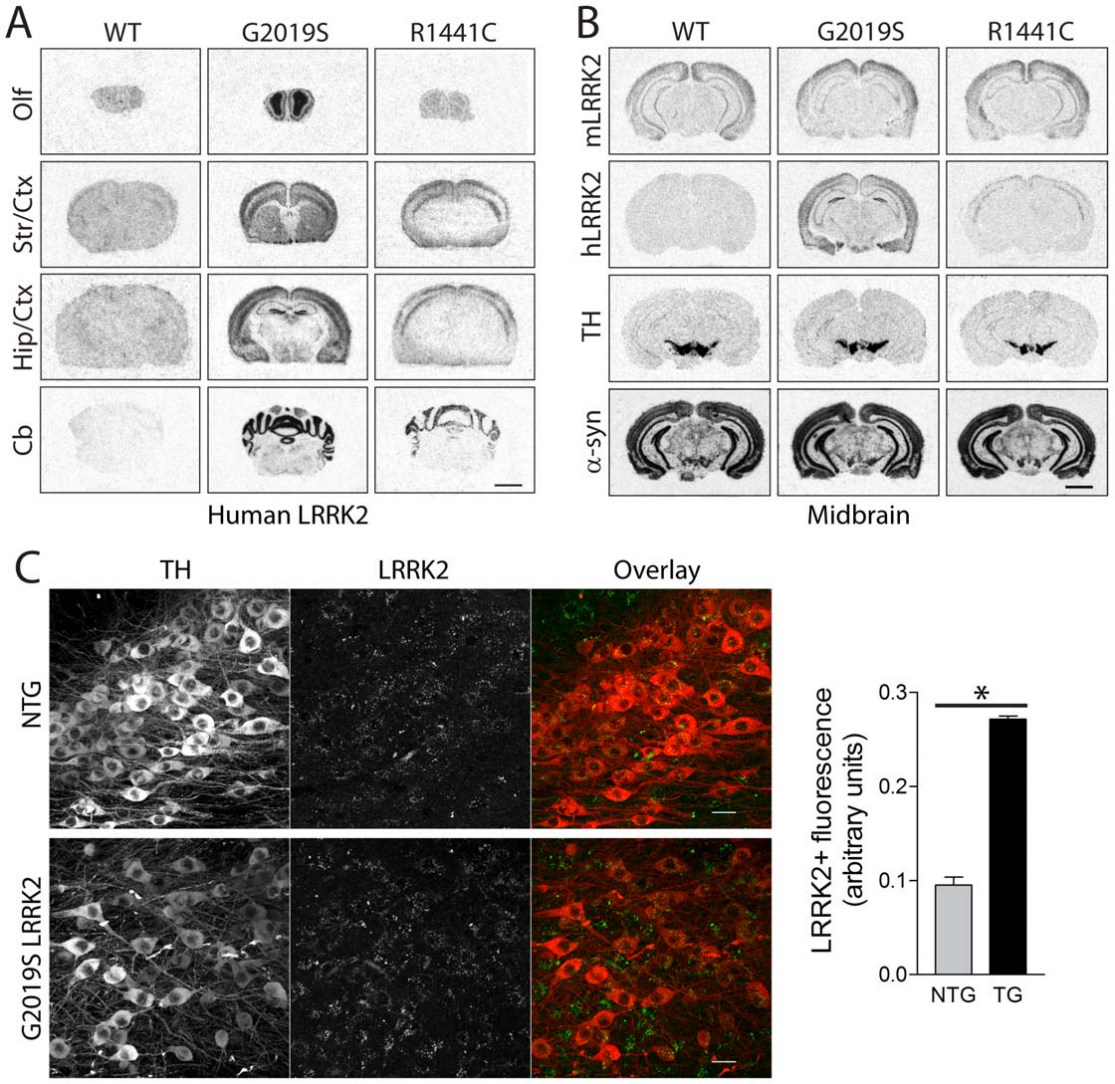
Localization of human LRRK2 in the brain of transgenic mice. ***A***, *In situ* hybridization with ^33^P-labeled antisense oligonucleotide probes specific to human LRRK2 mRNA. Autoradiographs of human LRRK2 in WT (line 249), G2019S (line 340) and R1441C (line 574) transgenic mice at 2–3 months, at the level of the olfactory bulb (*Olf*), striatum/cortex (*Str/Ctx*), hippocampus/cortex (*Hip/Ctx*) and cerebellum (*Cb*). ***B***, Localization of LRRK2 (mouse or human), and endogenous TH or α-synuclein mRNAs for comparison in adjacent midbrain sections of 2–3 month-old WT, G2019S and R1441C LRRK2 transgenic mice. ***C***, Confocal microscopic images of LRRK2 (mouse + human; MJFF2/c41-2 antibody) and tyrosine hydroxylase (TH) immunofluorescence in the substantia nigra of 4–5 month G2019S LRRK2 transgenic (TG) mice and their non-transgenic (NTG) littermates. Bar chart showing LRRK2+ fluorescence intensity localized within nigral TH+ dopaminergic neurons of TG and NTG mice. Bars present the mean ± SEM (*n* = 3 mice/genotype). *P<0.001 comparing TG and NTG as indicated. Scale bar: 25 µm (C).

We selected the highest expressing LRRK2 transgenic lines with similar protein levels for the R1441C (line 574) and G2019S (line 340) variants for further detailed analysis. WT-LRRK2 transgenic mice (line 249) express human LRRK2 mRNA and protein at lower levels than mutant LRRK2 lines and as such were only examined in some experiments (Figure 2A–B). The pattern of human LRRK2 mRNA expression was determined in the brains of transgenic mice by *in situ* hybridization with oligonucleotide probes (Figure 2A–B and S2). G2019S-LRRK2 mRNA is expressed throughout the mouse brain with highest expression in the olfactory bulb, cerebral cortex, hippocampus, striatum and cerebellum (Figure 2A) and clear expression in neurons of the substantia nigra pars compacta (Figure 2B and S2). We could further confirm the overexpression of G2019S LRRK2 protein (∼2.7-fold over endogenous LRRK2 levels) specifically within tyrosine hydroxylase (TH)-positive dopaminergic neurons of the substantia nigra pars compacta from transgenic mice by confocal fluorescence microscopy using a pan-LRRK2 antibody that detects both mouse and human LRRK2 (Figure 2C). The expression pattern of human G2019S-LRRK2 mRNA is broadly similar to endogenous LRRK2 mRNA in the mouse brain (Figure 2A–B and S3A). In general, human LRRK2 expression does not influence the expression level or pattern of endogenous LRRK2 mRNA in the brain, spleen or kidney of transgenic mice (Figure S3). Unexpectedly, R1441C-LRRK2 mRNA expression is detected at highest levels in the cerebral cortex and cerebellum but is not appreciably expressed in the striatum, hippocampus or ventral midbrain of transgenic mice (Figure 2A–B). Similarly, WT LRRK2 mRNA is widely expressed in the brains of transgenic mice but at markedly lower levels than G2019S LRRK2 mRNA and not appreciably within the ventral midbrain (Figure 2A–B). Therefore, only G2019S LRRK2 transgenic mice appear to be useful for assessing the impact of human LRRK2 expression on the nigrostriatal dopaminergic pathway. In general, LRRK2 transgenic mice are viable, fertile and produce normal numbers of progeny. LRRK2 mice are generally unremarkable with no obvious behavioral abnormalities, and no differences in body weight or survival compared to their non-transgenic littermates up to 24 months of age (data not shown).

### Progressive Dopaminergic Neuronal Loss in G2019S LRRK2 Transgenic Mice

**Figure 3:**
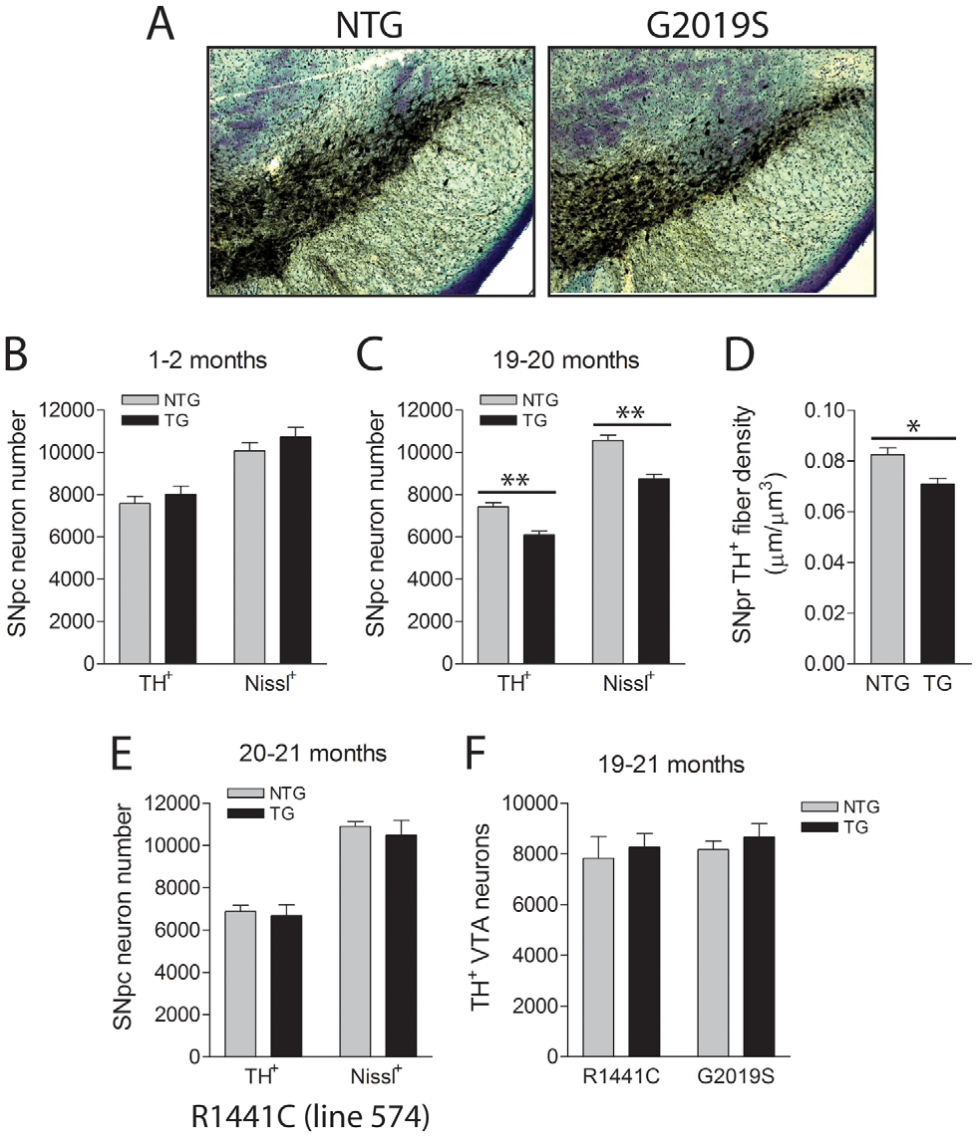
Progressive loss of substantia nigra dopaminergic neurons in G2019S LRRK2 transgenic mice. ***A***, Example of TH and Nissl staining in the substantia nigra of NTG and G2019S LRRK2 TG mice (line 340) at 19–20 months. ***B*** and ***C***, Stereological counts for TH+ and Nissl+ neurons in the pars compacta region of NTG and G2019S LRRK2 TG mice at (***B***) 1–2 months (*n* = 7–8 mice/genotype) and (***C***) 19–20 months (*n* = 5 mice/genotype). ***D***, Stereological measurement of TH+ dopaminergic neuritic density in the pars reticulata of NTG or TG G2019S mice at 19–20 months, expressed as average length of TH+ fibers (µm) per µm^3^ section area (*n* = 5–6 mice/genotype). ***E***, Stereological counts of TH+/Nissl+ neurons in the pars compacta of 20–21 month NTG or R1441C LRRK2 TG mice (line 574, *n* = 6–8 mice/genotype). ***F***, Stereological counts of TH+ neurons in the VTA region of 19–21 month R1441C or G2019S TG mice and their NTG littermates (*n* = 5 mice/genotype for G2019S or *n* = 7–8 for R1441C). Bars present the mean ± SEM. *P<0.02 and **P<0.005 comparing TG with NTG as indicated.

To determine whether the expression of G2019S-LRRK2 in mice induces the degeneration of nigrostriatal dopaminergic neurons with age, cohorts of LRRK2 transgenic mice were aged to 19–21 months. The numbers of TH+ and Nissl+ neurons in the substantia nigra pars compacta were counted using unbiased stereological methods (Figure 3). Remarkably, G2019S-LRRK2 mice exhibit a significant ∼18% loss of TH+ dopaminergic neurons and a corresponding ∼17% loss of Nissl+ nigral neurons compared to their non-transgenic littermates (Figure 3C), indicating dopaminergic neuronal degeneration rather than a loss of dopaminergic phenotype. At 1-2 months, G2019S-LRRK2 mice display normal numbers of TH+ and Nissl+ nigral neurons suggesting that neuronal loss occurs in a progressive manner (Figure 3B). We also observe a corresponding significant ∼14% reduction of TH+ dopaminergic neuritic density in the adjacent substantia nigra pars reticulata of 19–20 month-old G2019S-LRRK2 mice compared to their non-transgenic littermates (Figure 3D). The loss of dopaminergic neuritic density could result directly from the loss of dopaminergic neurons and/or a reduction in neuritic complexity. As expected, R1441C-LRRK2 mice display a normal number of nigral TH+ and Nissl+ neurons at 20–21 months (Figure 3E) consistent with the observed lack of transgene expression in the substantia nigra in this mouse line (Figure 2B). Furthermore, dopaminergic neuronal loss is not observed in a second lower-expressing G2019S-LRRK2 line (line 1128) at a similar advanced age implying that neurodegeneration in the 340 mouse line is most likely due to higher transgene expression (Figure S4). Two WT-LRRK2 transgenic mouse lines (lines 249 and 27) and a second R1441C-LRRK2 line (line 546) also fail to reveal dopaminergic neuronal loss (Figure S4) consistent with lower expression levels and/or restricted expression patterns of human LRRK2 in these mouse lines (Figure 1C and 2A–B). These observations would suggest that dopaminergic neurodegeneration in aged G2019S-LRRK2 mice (line 340) results from higher levels of expression of human LRRK2 directly in nigral dopaminergic neurons that may reach a critical threshold necessary for degeneration. To address the selective vulnerability of nigral dopaminergic neuronal loss in G2019S-LRRK2 mice, TH+ neurons were also counted in the adjacent ventral tegemental area (VTA) which contains a similar population of dopaminergic neurons projecting to the nucleus accumbens (mesolimbic pathway) and the frontal cortex (mesocortical pathway). G2019S- and R1441C-LRRK2 transgenic mice reveal normal numbers of TH+ dopaminergic VTA neurons at 19–21 months despite detectable expression of G2019S-LRRK2 mRNA in VTA neurons (Figure 3F and S2). Unexpectedly, the density of TH+ dopaminergic nerve terminals in the striatum of G2019S-LRRK2 mice at 19–20 months is normal compared to their non-transgenic littermates, which suggests a compensatory re-sprouting of the remaining dopaminergic neuronal processes (Figure S5). Collectively, our data demonstrates that G2019S-LRRK2 transgenic mice exhibit a progressive and relatively selective degeneration of nigrostriatal dopaminergic neurons.

### Dopamine Levels in G2019S LRRK2 Transgenic Mice

**Figure 4:**
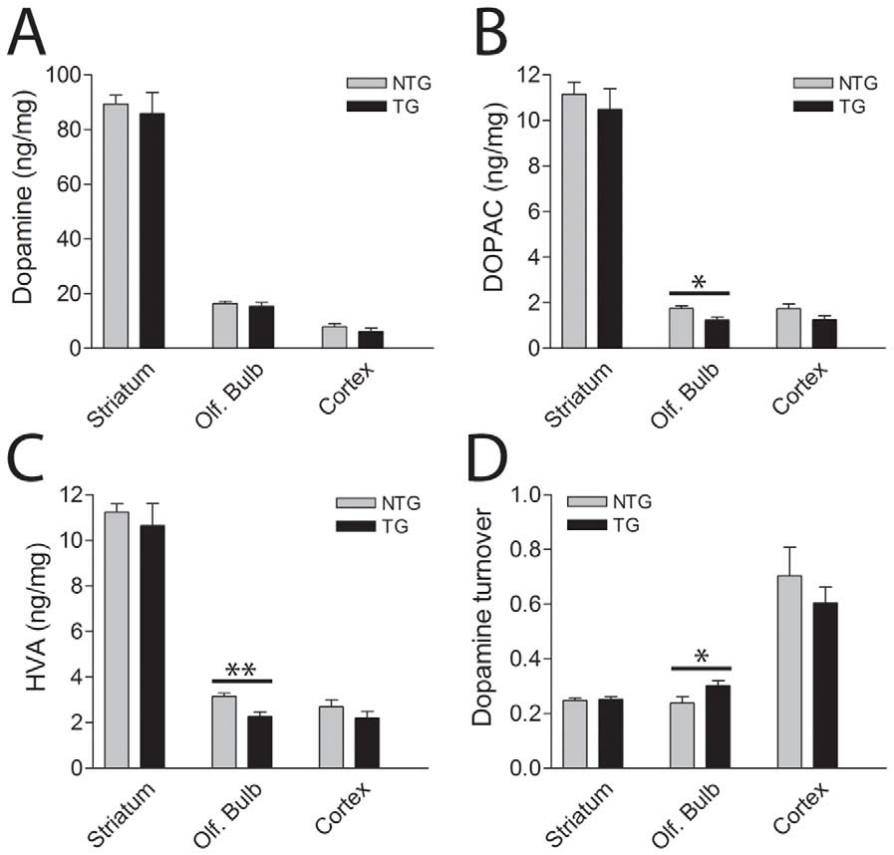
HPLC analysis of dopamine and its metabolites in G2019S LRRK2 transgenic mice. ***A–D***, Levels of (***A***) dopamine (DA) and its metabolites, (***B***) DOPAC and (***C***) HVA, and (***D***) dopamine turnover ([DOPAC+HVA]/DA) in the striatum, olfactory bulb and cerebral cortex of 14–15 month-old G2019S LRRK2 TG mice (line 340) and their NTG littermates by HPLC analysis (*n* = 8/genotype). Bars present the mean ± SEM. *P<0.05 or **P<0.005 comparing TG with NTG as indicated.

To further examine the impact of G2019S mutant LRRK2 expression on the nigrostriatal dopaminergic pathway, the levels of striatal dopamine and its metabolites, 3,4-dihydroxyphenylacetic acid (DOPAC) and homovanillic acid (HVA), were measured by HPLC analysis (Figure 4). At 14–15 months of age, G2019S-LRRK2 mice reveal normal levels of dopamine, DOPAC and HVA and normal dopamine turnover in the striatum and cerebral cortex (Figure 4A–D). This finding is largely consistent with the normal density of striatal dopaminergic nerve terminals at 19–20 months of age (Figure S5). Sufficient cohorts of G2019S-LRRK2 mice were not available to assess striatal dopamine content at later ages that parallel nigral dopaminergic cell loss. In the olfactory bulb, the levels of DOPAC and HVA are significantly reduced resulting in a modest enhancement of dopamine turnover compared to non-transgenic littermates (Figure 4A–D). Notably, G2019S-LRRK2 mRNA is expressed at highest levels in the olfactory bulb relative to the striatum or cerebral cortex (Figure 2A). G2019S-LRRK2 mice also exhibit a small yet significant increase in the levels of serotonin (5-HT) and its metabolite, 5-HIAA, in the prefrontal cortex (Figure S6A). As expected, the levels of striatal dopamine, DOPAC and HVA are normal in 19–20 month-old R1441C-LRRK2 mice (Figure S6B) consistent with the absence of detectable transgene expression in the nigrostriatal pathway (Figure 2B). However, aged R1441C-LRRK2 mice exhibit a significant reduction of cortical dopamine, DOPAC, HVA and norepinephrine (NE) levels compared to their non-transgenic littermates (Figure S6B) consistent with most prominent expression of R1441C-LRRK2 mRNA in the cerebral cortex (Figure 2A). The reduction of cortical catecholamine levels induced by R1441C-LRRK2 expression may indicate abnormalities in mesocortical dopaminergic neurotransmission. WT-LRRK2 mice (line 249) exhibit normal levels of dopamine, DOPAC and HVA in the striatum, cerebral cortex and olfactory bulb at 16–17 months of age (Figure S6C). Taken together, G2019S-LRRK2 mice reveal modestly enhanced dopamine turnover in the olfactory bulb but normal levels of striatal dopamine and its metabolites, whereas R1441C-LRRK2 mice reveal reduced levels of cortical catecholamines.

### Normal Locomotor Activity and Prepulse Inhibition in G2019S LRRK2 Transgenic Mice

**Figure 5:**
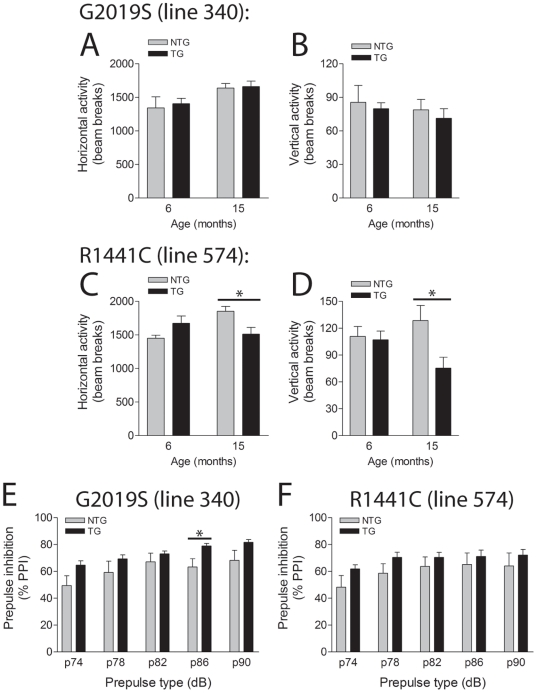
Behavioral analysis of LRRK2 transgenic mice. ***A–D***, In the open field G2019S LRRK2 TG mice (line 340) exhibit normal horizontal (***A***) and vertical (***B***) locomotor activity compared to NTG littermates at 6 and 15 months (*n* = 7–9 mice/genotype). R1441C LRRK2 TG mice (line 574) reveal normal locomotor activity at 6 months but deficits at 15 months (***C*** and ***D***) compared to NTG mice (*n* = 6-9 mice/genotype). Data represent the number of beam breaks during the first 15 min period. ***E–F***, Normal pre-pulse inhibition (PPI) of the acoustic startle response in LRRK2 transgenic mice. ***E*** and ***F***, G2019S and R1441C LRRK2 TG mice display normal PPI of the acoustic startle reflex at 15 months compared to their NTG littermates, with increasing pre-pulse tones of 74–90 dB (*n* = 6–8 mice/genotype). Data are expressed as % PPI relative to no pre-pulse tone. Bars present the mean ± SEM. *P<0.05 comparing TG with NTG as indicated.

To investigate whether G2019S mutant LRRK2 expression influences motor performance, we measured locomotor activity in the open field quadrant (Figure 5). G2019S-LRRK2 mice display normal locomotor activity in the open field at 6 and 15 months of age (Figure 5A–B). In contrast, R1441C-LRRK2 mice exhibit a significant reduction in horizontal and vertical locomotor activity at 15 months compared to their non-transgenic littermate mice that is not evident at 6 months (Fig. 5C–D). We also assessed prepulse inhibition of the acoustic startle reflex, a measure of sensorimotor gating that can be modulated in part by dopaminergic neurotransmission [39]. However, G2019S- and R1441C-LRRK2 transgenic mice do not perform differently from their non-transgenic littermates when tested at 6 months (data not shown) and 15 months of age (Figure 5E–F). Our data demonstrate that G2019S-LRRK2 expression does not influence locomotor activity or prepulse inhibition of the acoustic startle reflex in aged mice. Furthermore, our data suggests that R1441C-LRRK2 mice exhibit a progressive impairment of locomotor activity.

### Reduced Complexity of Dopaminergic Neurites in Primary Midbrain Cultures from G2019S LRRK2 Transgenic Mice

**Figure 6:**
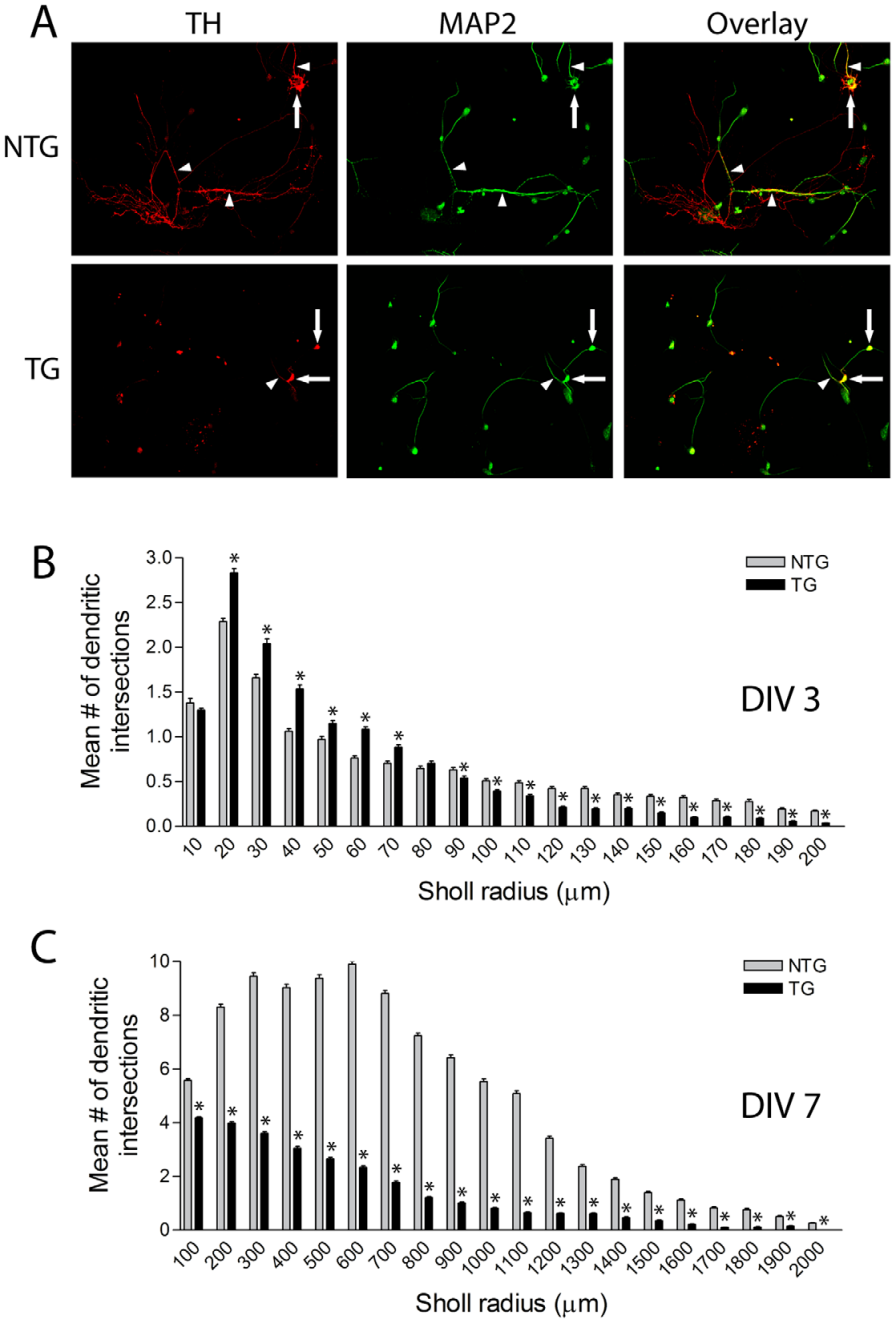
Reduced neuritic complexity of G2019S LRRK2 dopaminergic neurons *in vitro*. ***A***, Example of neuritic morphology of immunoreactive TH+ and MAP+ dopaminergic neurons in primary midbrain cultures derived from G2019S LRRK2 TG mice (line 340) and their NTG littermates at DIV 7. TH+ neuronal soma (*arrows*) and neurites (*arrowheads*) are indicated. ***B–C***, Sholl analysis of TH+ dopaminergic neurites plotting the mean number of dendritic intersections with circles of increasing radii at DIV 3 (***B***) and DIV 7 (***C***). Data represents the mean number of dendritic intersections within each circular interval (µm) from independent cultures derived from 4 mice per genotype. Bars present the mean ± SEM (DIV 3: NTG, *n* = 94 and TG, *n* = 150; DIV 7: NTG, *n* = 54 and TG, *n* = 71 neurons). *P<0.05 comparing TG with NTG as indicated.

LRRK2 has been shown to regulate the morphology of neuronal processes in cultured primary cortical neurons [20], [40]. To further explore this phenotype in our LRRK2 transgenic mice, primary midbrain cultures were prepared from the ventral mesencephalon of G2019S-LRRK2 transgenic mice (line 340) and their non-transgenic littermates. These cultures typically contain 5–10% of TH+ dopaminergic neurons (unpublished observation). Sholl analysis was conducted to provide a measure of neuritic complexity (Figure 6 and Table 1). At *days-in-vitro* (DIV) 3, developing dopaminergic neurons from G2019S-LRRK2 mice exhibit significantly reduced overall neurite complexity manifesting as shorter neurites but with modestly increased neurite branching compared to non-transgenic neurons (Figure 6B and Table 1). However, at DIV 7 when dopaminergic neurite outgrowth is fully established, G2019S-LRRK2 dopaminergic neurons reveal a dramatic reduction of neurite length and branching with an overall reduction in neurite complexity (Figure 6A and 6C, Table 1). We did not perform similar measurements on cultured dopaminergic neurons derived from R1441C-LRRK2 mice (line 574) since these transgenic mice do not exhibit transgene expression within the nigrostriatal dopaminergic pathway (Figure 2B). Collectively, our data demonstrate that G2019S-LRRK2 expression dramatically reduces the neuritic complexity of cultured dopaminergic neurons.

**Table 1:** Sholl analysis of TH+ dopaminergic neurons from G2019S LRRK2 mice (line 340).

Type of measurement	Definition	DIV	NTG	G2019S
*Sholl coefficient* (-log slope of regression fit)	Overall measure of dendritic complexity	3	−0.0305±0.0022	-0.0385±0.0018*
		7	−0.0026±0.0002	−0.0075±0.0001**
*Schoenen ramification index*	Av. density of branching per primary dendrite	3	1.14±0.12	1.42±0.12**
		7	3.84±0.31	1.98±0.15**
*Rmax* (µm)	Av. radius where neuritic intersections are maximal	3	19.71±2.92	15.10±1.19
		7	482.03±47.61	176.05±27.40**
*Max. neurite length* (µm)	Av. radius where longest neurite extends to	3	111.46±11.50	87.19±7.12*
		7	1326.72±64.53	606.10±55.23**

*P<0.02,

**P<0.001 versus NTG. *Abbreviations:* DIV, days in vitro; NTG, non-transgenic; Av., average.

### Autophagic Abnormalities in G2019S LRRK2 Transgenic Mice

*LRRK2*-linked PD is associated with heterogeneous neuropathology, including Lewy bodies, neurofibrillary tau pathology, ubiquitin-positive inclusions or in some cases the absence of inclusions [3], [10], [41]. To investigate pathology in G2019S-LRRK2 mice (line 340), immunohistochemistry with a number of pathological markers was conducted in the ventral midbrain, striatum and cerebral cortex at 23–24 months of age. G2019S-LRRK2 mice do not reveal abnormalities in the distribution of α-synuclein, ubiquitin, tau and GFAP compared to their non-transgenic littermates (Figure S7), and do not exhibit abnormal staining for phospho-α-synuclein (pS^129^) or phospho-tau (pS^396^/pS^404^) (Figure S7).

**Figure 7:**
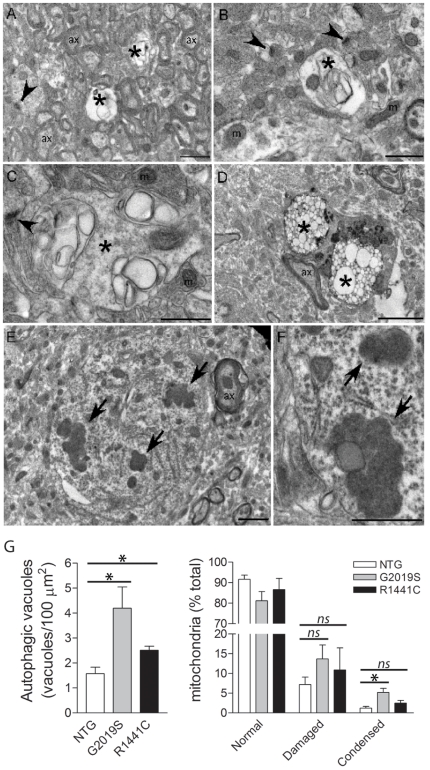
Transmission electron microscopic (TEM) analysis of LRRK2 transgenic mice. TEM analysis of cerebral cortex tissue from 17–18 month G2019S LRRK2 transgenic mice (line 340). ***A–D***, Vacuoles with multiple membranes resembling autophagosomes or autophagic vacuoles (indicated by *) are observed within regions enriched in axons and/or synapses (***A–C***) or within neuronal soma (***D***). ***E*** and ***F***, Clusters of condensed mitochondria within the neuronal soma (indicated by arrows) reminiscent of mitochondria that are undergoing autophagocytosis. Synapses (*arrowheads*), axons (*ax*) and normal mitochondria (*m*) are indicated. ***G***, Quantitation of the density of autophagic vacuoles and the proportion of normal, damaged or condensed/aggregated mitochondria in equivalent regions of cingulate cortex from 17–26 month G2019S and R1441C LRRK2 transgenic (TG) mice relative to their non-transgenic (NTG) littermates. Bars represent the mean ± SEM (*n* = 3 mice/genotype). *P<0.05 comparing TG and NTG mice as indicated. Scale bars: 2 µm (A, E, F) or 1 µm (B–D).

Transmission electron microscopy (TEM) was performed on the cerebral cortex and striatum from G2019S-LRRK2 mice (line 340) at 17–18 months to investigate more subtle pathological abnormalities. Cytopathological abnormalities are observed in G2019S-LRRK2 mice including enlarged vacuolar structures with multiple membranes resembling autophagic vacuoles including early and late autophagosomes present in neuronal soma and regions enriched for axons and synapses (Figure 7A–D and S8). Autophagic vacuoles are frequently observed within neuronal soma and axonal processes (Figure S9). We also observe condensed aggregated mitochondria in neuronal soma consistent with increased mitochondrial autophagy, in addition to damaged mitochondria (Figure 7E–G and S10). Similar yet less pronounced cytopathology is observed in the cortex of R1441C-LRRK2 mice (line 574) at 20–23 months (data not shown). Quantitation of TEM cytopathology within the cortex reveals that G2019S LRRK2 transgenic mice exhibit a significant increase in the density of autophagic vacuoles and the proportion of abnormal condensed mitochondria compared to their non-transgenic littermates (Figure 7G). R1441C LRRK2 mice also display a smaller yet significant increase in the density of autophagic vacuoles (Figure 7G). A significant accumulation of autophagic vacuoles is also evident in the striatum of G2019S LRRK2 mice (Figure S8). Our data demonstrate that G2019S- and R1441C-LRRK2 transgenic mice exhibit autophagic abnormalities in the brain with advanced age.

## Discussion

The major finding of this study is the observation that the expression of human G2019S LRRK2 induces the progressive degeneration of nigrostriatal pathway dopaminergic neurons *in vivo* in transgenic mice. Accompanying the loss of dopaminergic neurons are autophagic and mitochondrial abnormalities throughout the mouse brain, as revealed by electron microscopy, and reduced neurite complexity of cultured midbrain dopaminergic neurons. G2019S LRRK2 expression in aged mice, however, fails to influence the levels of striatal dopamine and its metabolites, affect locomotor activity or produce abnormal protein inclusion pathology. In contrast, aged R1441C LRRK2 transgenic mice with a restricted pattern of transgene expression display reduced levels of cortical catecholamines, a progressive impairment of locomotor activity and the accumulation of autophagic vacuoles in the cerebral cortex. Collectively, our study reveals a number of intriguing phenotypes caused by the expression of LRRK2 harboring the PD-associated G2019S and R1441C mutations *in vivo*. Importantly, similar to *LRRK2*-linked PD, expression of G2019S mutant LRRK2 is sufficient to precipitate modest nigral dopaminergic neuronal loss with advanced age.

It is unlikely that a ∼20% loss of dopaminergic neurons would be sufficient to produce motor impairment or striatal dopamine deficits in aged G2019S LRRK2 mice especially since the density of dopaminergic striatal nerve terminals appears normal at this age possibly due to the compensatory re-sprouting of existing nerve terminals. We do not observe dopaminergic neuronal loss in five additional LRRK2 transgenic lines (WT, R1441C or G2019S) at similar advanced ages. The neuronal loss in G2019S LRRK2 mice (line 340) most likely reflects higher levels of transgene expression and detectable expression in dopaminergic neurons of the substantia nigra in this mouse line. The loss of nigral dopaminergic neurons is also relatively selective as dopaminergic neurons of the adjacent VTA (A10 nucleus) are spared despite expression of G2019S LRRK2 in this population. As our LRRK2 transgenic lines do not share matched transgene expression within the substantia nigra, it is difficult to determine whether the equivalent expression of WT or R1441C mutant LRRK2 would also be sufficient to precipitate neuronal loss similar to G2019S LRRK2. While we could clearly demonstrate the expression of G2019S LRRK2 in nigral dopaminergic neurons, this was not possible for WT or R1441C LRRK2 mice. A further caveat is that WT and R1441C LRRK2 are generally expressed at lower levels than G2019S LRRK2 in the brains of transgenic mice. At this juncture it is unclear whether nigral dopaminergic degeneration in G2019S LRRK2 transgenic mice is a result of the pathogenic actions of the G2019S mutation or is due to the overexpression of human LRRK2. That five additional LRRK2 transgenic lines with varying transgene expression patterns and levels do not display nigral neuronal loss perhaps suggests that human LRRK2 overexpression *per se* is not sufficient to precipitate neuronal degeneration. BAC and inducible transgenic mouse models overexpressing human LRRK2 variants also do not display nigral neuronal loss although it is unclear to what degree transgenes in these mice are expressed in substantia nigra dopaminergic neurons [32]–[34]. Further supporting the pathogenicity of the G2019S mutation in our transgenic mice are the recent observations that viral-mediated expression of human G2019S LRRK2 in the substantia nigra dopaminergic neurons of rodents causes the progressive degeneration of these neurons whereas the equivalent expression of WT LRRK2 or GFP failed to induce neuronal degeneration [42], [43]. These observations support a specific pathogenic effect of G2019S LRRK2 on nigral dopaminergic neurons in our LRRK2 transgenic mice.

While certain mutant LRRK2 BAC transgenic models exhibit subtle tau pathology or processing abnormalities in the absence of frank neuronal loss [31]–[32], [34], our G2019S LRRK2 mice lack obvious protein inclusion pathology that is observed in PD brains bearing *LRRK2* mutations, including α-synuclein, tau or ubiquitin inclusions [10]. This finding suggests that dopaminergic neuronal degeneration in our G2019S LRRK2 model may occur independently from abnormal inclusion pathology or protein aggregation. We cannot exclude the possibility that submicroscopic protein aggregates contribute to neuronal loss or that inclusions are rapidly removed following neuronal death. In a rat LRRK2 adenoviral model, we recently found that tau hyperphosphorylation in nigral dopaminergic neurites is induced equally by the expression of WT and G2019S human LRRK2 in a transient manner, whereas only G2019S LRRK2 expression induced dopaminergic neuronal loss. Thus, the induction of tau pathology and neuronal loss in dopaminergic neurons by human LRRK2 expression can be dissociated at least in this rat viral model [43]. Although *LRRK2* mutations are predominantly associated with Lewy body pathology in PD brains [44]–[45], some *LRRK2* mutations produce nigral degeneration without additional pathology [41] suggesting that protein aggregation may not be an absolute requirement for mutant LRRK2-induced neurodegeneration. The data obtained in our G2019S LRRK2 mice supports this assertion.

Coincident with neuronal loss in G2019S LRRK2 mice, we observe the accumulation of autophagic vacuoles and evidence of increased mitochondrial autophagy and damage in the brains of transgenic mice by electron microscopy. Of particular interest is that both R1441C and G2019S LRRK2 transgenic mice consistently display autophagic abnormalities in the cerebral cortex; a region where each transgene is prominently expressed in these models. At least *in vitro*, alterations in autophagy have been reported to modulate G2019S LRRK2-induced neurite shortening and the expression of R1441G LRRK2 leads to the accumulation of autophagic vacuoles and multivesicular bodies [23], [46]. In a yeast model of LRRK2-induced cytotoxicity there is an accumulation of autophagic vacuoles together with associated defects in endocytic vesicular trafficking [47]. Overexpression of G2019S LRRK2 in cultured neurons induces the accumulation of swollen lysosomes, multivesicular bodies, distended vacuolated mitochondria and phosphorylated tau-positive spheroid axonal inclusions [20]. Taken together, our data demonstrate for the first time that mutant LRRK2 expression *in vivo* can cause abnormalities in the autophagy pathway. It is not clear at present whether the abnormal accumulation of autophagic vacuoles induced by G2019S and R1441C LRRK2 expression *in vivo* reflects the impairment or activation of the autophagy pathway, or instead manifests through impaired autophagic flux due to the downstream inhibition of autolysosome formation and/or lysosomal function. Whether or not such autophagic alterations underlie dopaminergic neuronal death in G2019S LRRK2 transgenic mice is unclear but certainly warrants further attention. At least in cultured neuronal cells, G2019S LRRK2-induced neurite shortening is mediated, in part, by the autophagy pathway and can be exacerbated by activation of autophagy through treatment with rapamycin [23]. It will be important to determine whether similar vesicular abnormalities are also observed in PD brains with *LRRK2* mutations.

G2019S LRRK2 expression reduces the neuritic complexity of cultured dopaminergic neurons derived from G2019S LRRK2 transgenic mice. A similar reduction in neurite length and branching of cortical neurons has also been demonstrated *in vitro* following expression of mutant LRRK2 [20], [40], [48]. In cultured neurons, the reduced neuritic complexity results from the reduced outgrowth of neurites rather than from neurite retraction since the neuritic phenotype is already apparent in developing neurons before neurites have fully extended [40]. However, the reduced complexity of dopaminergic neurites would appear to be a phenotype specific to cultured neurons since we find no evidence for abnormal dopaminergic neurons in the brains of young G2019S LRRK2 mice. Alterations in neuritic complexity may become apparent due to the rapid neurite outgrowth experienced by neurons under culture conditions, a situation which is not likely mimicked *in vivo*. It is not yet clear whether reduced neuritic complexity is a precursor to neuronal death following LRRK2 overexpression or whether this neuritic phenotype reflects an independent physiological function of LRRK2 in regulating neuritic morphology.

Together, our data provide evidence that expression of human LRRK2 harboring the G2019S mutation *in vivo* is sufficient to recreate the slowly progressive degeneration of dopaminergic neurons that forms the hallmark pathology of familial and sporadic PD. LRRK2 transgenic mice fail, however, to reveal additional key phenotypes related to *LRRK2*-linked or sporadic PD supporting previous observations that mouse models based upon monogenic causes of familial PD are not sufficient alone to recapitulate the full spectrum of disease [49]. Our study reveals a potential role for alterations in autophagy and neuritic morphology in mediating the pathogenic effects of *LRRK2* mutations *in vivo*. The mouse models presented here together with similar recent studies [31]–[35] reveal a role for familial *LRRK2* mutations in mediating the dysfunction of the nigrostriatal dopaminergic pathway. The current LRRK2 mouse models will provide important tools for understanding the mechanism(s) through which familial mutations precipitate neuronal degeneration and PD.

## Materials and Methods

### Animals

Mice were housed and treated in strict accordance with the NIH *Guide for the Care and Use of Laboratory Animals*. All animal procedures were approved by the Institutional Animal Care and Use Committees of the Johns Hopkins Medical Institutions (Animal Welfare Assurance No. A3272-01), or were conducted in accordance with the Swiss legislation (Canton de Vaud, Animal Authorization No. 2293) and the European Community Council directive (86/609/EEC) for the care and use of laboratory animals. Mice were maintained in a pathogen-free facility and exposed to a 12 h light/dark cycle with food and water provided *ad libitum*.

### Generation of LRRK2 transgenic mice

To generate LRRK2 transgenic mice, we used a pGL3-Basic vector (Promega, Madison, WI, USA) containing the ∼1.5 kb human PDGFβ promoter preceded by a ∼400 bp CMV immediate-early enhancer inserted between 5′ *Kpn*I and 3′ *Hind*III sites. The pGL3-CMVE-PDGFβ construct was kindly provided by Dr. S. Wang (National University of Singapore, Singapore) [36]. The *Hind*III site was converted to a *Nhe*I site by digestion, Klenow fill-in and blunt-end ligation. A ∼200 bp chimeric intron from vector pCI (Promega) was inserted into the *Nhe*I site. A luciferase cDNA was removed by digestion with *Nhe*I and *Xba*I and replaced by a *Nhe*I-flanked human LRRK2 cDNA. The ∼7.6 kb LRRK2 cDNA from a pcDNA3.1-LRRK2-Myc-His vector [18] was previously modified to introduce a 5′ Kozak sequence and 3′ stop codon (TAA) between flanking *Nhe*I sites. A ∼10.5 kb transgene cassette was excised by digestion with *Kpn*I and *Drd*I and purified by GELase extraction (Epicentre Biotechnologies, Madison, WI, USA). Transgene DNA was microinjected into the pronucleus of single-cell embryos (C57BL/6J x C3H/HeJ F1 hybrids) and implanted into pseudo-pregnant female mice. Founder mice were identified by genomic PCR with transgene-specific primers flanking the CMVE-PDGFβ region (P1, 5′-ATTACCATGGTTCGAGGTGA-3′ and P2, 5′-CAAGTGTCTGCAGGAAGGTT-3′) and the LRRK2-SV40 region (P3, 5′-TGGTTTGTCCAAACTCATCA-3′ and P4, 5′-CGTTTGGGACATCAATCTTC-3′) producing a ∼170 bp product together with mouse GAPDH primers (F1, 5′-TGTTTGTGATGGGTGTGAAC-3′ and R1, 5′-TACTTGGCAGGTTTCTCCAG-3′) producing a ∼380 bp internal control. Founder mice were bred with C57BL/6J mice to produce F1 hemizygous mice for expression analysis. In general, LRRK2 transgenic mice were maintained as hemizygotes by backcrossing to the C57BL/6J strain for 3-4 generations.

For RT-PCR, total RNA was purified from mouse hemi-brains using an RNeasy kit (Qiagen, Valencia, CA, USA) and digested with DNase I (Qiagen). RNA was reverse transcribed using a SuperScript III First-Strand Synthesis system (Invitrogen, Carlsbad, CA, USA) with oligo(dT)_20_. cDNA derived from 50 ng of total RNA was amplified by PCR with primers specific to human LRRK2 (TGLRK3-F, GAAGATTGATGTCCCAAACG and TGLRK3-R, GAACTGGAGGAGGCCATATT) or β-actin (sense, 5′-GCTCGTCGTCGACAACGGCTC-3′, and antisense, 5′-CAAACATGATCTGGGTCATCTTCTC-3′) producing products of 220 bp and 353 bp, respectively. For semi-quantitative analysis, increasing PCR cycles and densitometry were used to construct PCR amplification curves to identify the exponential phase. For each PCR, 30 cycles for LRRK2 and 25 cycles for β-actin were compared for semi-quantitative analysis.

### In situ hybridization

*In situ* hybridization was performed as previously described [50], [51] on fresh-frozen brain sections from 2-3 month-old mice using species-specific ^33^P-labeled oligonucleotide probes complementary to human LRRK2 (nt 4436-4483; NM_198578.3), mouse LRRK2 (nt 6036-6085; NM_025730.2), mouse TH (nt 197-248; NM_009377.1) or mouse α-synuclein (nt 562-611; NM_00104245.1). mRNA signals were revealed by exposure of slides to autoradiographic film or by development in photo-emulsion for microscopic inspection.

### Western blot analysis

Protein extracts were prepared from hemi-brains of 2-4 month-old mice as previously described [52] by homogenization in TNE buffer (10 mM Tris-HCl pH 7.4, 150 mM NaCl, 5 mM EDTA, 0.5% NP-40, 1 X Complete protease inhibitor cocktail [Roche], 1 X phosphatase inhibitor cocktail I and II [Sigma-Aldrich, St. Louis, MO, USA]). Protein concentration was determined by BCA method (Pierce Biotechnology, Rockford, IL, USA) and 75 µg of protein was resolved by SDS-PAGE, transferred to nitrocellulose and probed with rabbit anti-LRRK2 antibodies recognizing human/mouse (JH5514 [53] or monoclonal c81-8, Epitomics, Inc, Burlingame, CA) or human-specific (NB300-267, Novus Biologicals, Littleton, CO, USA [54]) epitopes, or mouse β-tubulin (TUB 2.1, Sigma-Aldrich) antibody. Refer to www.pdonlineresearch.org for detailed characterization of rabbit monoclonal LRRK2 antibody (clone c81-8, Epitomics, Inc). Quantitation of total LRRK2 and β-tubulin protein levels by densitometry was conducted using LabImage 1D software (Kapelan Bio-Imaging Solutions, Leipzig, Germany) on Western blot images captured using a FujiFilm LAS-4000 Luminescent Image Analysis system.

### Measurement of biogenic amines by HPLC

HPLC with electrochemical detection was employed to measure the concentration of the biogenic amines, dopamine (DA), 3,4-dihydroxyphenylacetic acid (DOPAC), homovanillic acid (HVA), 5-hydroxytryptamine (5-HT), 5-hydroxyindoleacetic acid (5-HIAA) and norepinephrine (NE) as previously described [55]. Samples were prepared from dissected brain regions and injected onto a C-18 80×4.6 mm column (ESA Inc., Chelmsford, MA, USA) with detection using a 2-channel Coulochem II electrochemical detector (ES Inc.). Data were processed on an EZChrome Elite Client Workstation (ESA Inc.) with biogenic amine concentration expressed as ng per mg protein.

### TH immunohistochemistry and stereological assessments

#### TH/Nissl staining

Mice were anesthetized with sodium pentobarbital (150 mg/kg) before intracardial perfusion with cold PBS and 4% paraformaldehyde (PFA). Brains were removed, post-fixed with PFA overnight and cryopreserved in 30% sucrose in PBS at 4°C for 48 h. Coronal midbrain sections (40-µm) were prepared and processed for immunohistochemistry with rabbit anti-TH antibody (Novus Biologicals) and Nissl counterstain as previously described [55].

#### Stereological assessment of TH+/Nissl+ neurons

Unbiased stereological methodology was employed as described previously [55], [56] to count TH+ and Nissl+ neurons in the left and right pars compacta region of every fourth section throughout the ventral midbrain. Stereological counts were obtained using a computer-assisted image analysis system consisting of an Axiophot 2 photomicroscope (Carl Zeiss Inc., Thornwood, NY, USA) equipped with a computer-controlled motorized stage (Ludl Electronics, Hawthorne, NY, USA), a Hitachi HV C20 video camera, and interfaced with a StereoInvestigator system (MBF Bioscience, Williston, VT, USA) with optical fractionator probe. TH+ neurons in the VTA were counted by stereology in a similar manner. In general, we used a 40×40 µm counting frame, a 1 µm guard, 100×100 µm sampling grid, and a dissector height of 8 µm. Investigators were blinded to the genotype of each mouse.

#### Stereological assessment of TH+ fibers

To determine the length/density of TH+ fibers in the substantia nigra pars reticulata, we performed stereological length estimation with spherical probes on every fourth midbrain section, as previously described [57]. Briefly, virtual spherical probes were placed within a 40-µm thick section and the intersection of TH+ fibers with the sphere were counted. The lengths were measured at 50 random locations throughout the reference space. At each focal plane, concentric circles of progressively increasing and decreasing diameters were superimposed, and the intersections with the TH+ fibers and circles were counted (*Q*). To minimize surface artifacts, a guard volume of 1 µm was used. This method allows the determination of the total length density (*L_V_*) and the total length (*L*). To reduce the effects of variations in the area selection, *L_V_* was used for comparison between groups.

### Immunohistochemistry

Coronal sections (30-µm) from cortex, striatum and midbrain were prepared and processed for immunohistochemistry for various pathological markers as previously described [53], [54]. Briefly, sections were quenched of endogenous peroxidase activity, permeabilized, and incubated with primary antibodies for α-synuclein (Syn-1; BD Biosciences, San Jose, CA, USA), phospho-Ser129-α-synuclein (Wako Chemicals, Richmond, VA, USA), ubiquitin (DAKO, Carpinteria, CA, USA), tau (TAU-5; BD Biosciences), phospho-Ser396/404-tau (PHF1; kindly provided by Prof. Peter Davies, Albert Einstein College of Medicine, New York) and glial fibrillary acidic protein (GFAP; Sigma-Aldrich). Sections were processed with biotinylated anti-rabbit IgG or anti-mouse IgG antibodies and avidin-biotin-complex coupled to horse radish peroxidase (Vectastain ABC; Vector Laboratories, Burlingame, CA, USA), and visualized with 3,3′-Diaminobenzidine (DAB) reagent (Sigma-Aldrich).

### Immunofluorescence and confocal microscopy

Coronal midbrain sections (30 µm) were prepared from 4-5 month G2019S LRRK2 transgenic mice (TG; line 340) and non-transgenic littermate mice (NTG). Sections were processed for immunohistochemistry with rabbit monoclonal anti-LRRK2 antibody (clone c41-2, Epitomics; refer to www.pdonlineresearch.org for detailed characterization of this antibody) recognizing human and mouse epitopes, and mouse monoclonal anti-TH antibody (clone TH-2, Sigma-Aldrich), and anti-rabbit IgG-AlexaFluor-488 and anti-mouse IgG-AlexaFluor-633 antibodies (Invitrogen). Fluorescent images were captured using a Leica SP2 inverted confocal microscope in x, y and z planes. Relative LRRK2+ fluorescence intensity localized within substantia nigra TH+ neurons was determined using NIH ImageJ software by analysis of fluorescence signal in every fourth section throughout the entire midbrain region of each mouse.

### Transmission electron microscopy (TEM)

For TEM analysis, mice were perfused with fixative (3% PFA, 1.5% glutaraldehyde, 100 mM cacodylate, 2.5% sucrose, pH 7.4) and post-fixed for 1 h. Cortex and striatal sections were processed as described [58]. Sections were post-fixed in Palade's OsO_4_, *en bloc* stained in Kellenberger's uranyl acetate, dehydrated, and flat-embedded in epon. 80 nm *en face* sections were prepared on a Leica UCT ultramicrotome, collected onto 400 mesh high transmission nickel grids, and post-stained with lead and uranyl acetate. Images were collected on a Philips EM 410 TEM equipped with a Soft Imaging System Megaview III digital camera.

For quantitation of cytopathology, images were collected on a Philips FEI CM10 TEM equipped with a 11-megapixel Morada soft imaging system camera (Olympus) at the Interdisciplinary Centre for Electron Microscopy (CIME) at EPFL. TEM images were sampled at random over equivalent areas of 800×800 µm from representative regions of cingulate cortex. We analyzed between 50-100 images for each animal representing an average total area of 2348±323 µm^2^ per animal. The total number of autophagic vacuoles and mitochondria were measured within each image. The mean number of autophagic vacuoles per µm^2^ area was determined for LRRK2 transgenic (TG) and non-transgenic (NTG) mice. Mitochondria were subclassified as either morphologically normal (class I), damaged (class II) or abnormally condensed/aggregated based upon previously established criteria ([59] and refer to Figure S10). Class I and II mitochondria were distinguished based on the appearance of the electron transparent cristae with class II representing damaged pre-apoptotic mitochondria, whereas condensed mitochondria were identified as mitochondrial aggregates with condensed cristae. An average of 890±200 mitochondria were counted per animal. Mitochondrial subclasses were expressed as a percent of the total number of mitochondria for each animal.

### Primary midbrain cultures and analysis of neuritic complexity

Whole brains were dissected from P0 mice and the ventral mesencephalic region containing the substantia nigra (A9) and VTA (A10) was stereoscopically isolated and dissociated in media containing papain (20 U/ml). The cells were grown on coverslips pre-coated with mouse laminin (33 µg/ml; Invitrogen) and poly-*D*-lysine (20 ng/ml; Becton Dickinson) in media consisting of Neurobasal (Invitrogen), B27 supplement (2% w/v), L-glutamine (500 µM) and penicillin/streptomycin (100 U/ml). At DIV 3, the cells were treated with β-D-arabinofuranoside (10 µM) to inhibit glial cell division.

Cultures were fixed with 4% PFA and processed for immunocytochemistry with rabbit anti-TH (Novus Biologicals) and mouse anti-MAP2 (Sigma-Aldrich) antibodies and anti-rabbit IgG-AlexaFluor-546 and anti-mouse IgG-AlexaFluor-488 antibodies (Invitrogen). Fluorescent images were captured using a Leica DMI 4000 inverted fluorescence microscope (Leica) at 10x magnification. Sholl analysis was performed on TH+/MAP+ neurons using NIH ImageJ software with a Sholl analysis plug-in (v1.0) developed by the laboratory of Anirvan Ghosh (http://www.biology.ucsd.edu/labs/ghosh/software/index.html) [60] to quantify neuritic complexity. Briefly, we constructed continuous concentric circles centered upon the neuronal soma to measure the number of intersections of dendrites with circles of increasing radii. Sholl analysis was performed using the semi-log method and measurements are shown in Table 1.

### Behavioral analysis

#### Open field test

Novelty-induced locomotor activity in the open field quadrant was assessed over a 60 min period using four activity chambers with 16×16 infrared beams (San Diego Instruments, San Diego, CA, USA) [61]. Horizontal and vertical activities were automatically recorded. Activity recorded during the first 15 min period was used for analysis.

#### Acoustic startle response

Two identical startle chambers (San Diego Instruments) were used for measuring startle reactivity and plasticity as previously described [61]. Briefly, the experimental session consisted of a 5 min acclimatization period to a 70-dB background noise (continuous throughout the session), followed by a habituation session with the presentation of 10×40-ms 120-dB white noise stimuli at a 20-s inter-stimulus interval. Mice were left for 5 min in the chamber without acoustic stimuli and then subjected to the pre-pulse inhibition (PPI) session. For each PPI session, mice were exposed to various trials: 1) pulse-alone trial (120-dB, 100-ms broadband burst), 2) no stimuli trial, and 3) five prepulse-pulse combinations consisting of a 20-ms broadband burst used as a pre-pulse and presented 80-ms before the pulse (120 dB) using one of the five pre-pulse intensities (74, 78, 82, 86 and 90 dB). Each session consisted of six presentations of each type of trial presented in a pseudorandom order. PPI was calculated as % PPI: 100× (mean startle amplitude on pulse alone trials – mean startle amplitude on prepulse-pulse trials/mean startle amplitude on pulse alone trials) for each animal and data were plotted at mean % PPI for each prepulse type.

### Statistical analysis

Data were analyzed by two-tailed, unpaired Student's *t*-test by comparison of non-transgenic and transgenic mice for each condition or data interval. *P*<0.05 was considered significant.

## Supporting Information

Figure S1 Quantitation of LRRK2 protein levels in LRRK2 transgenic mice. Western blot analysis of soluble brain extracts (75 μg protein) derived from hemi-brains of 3-4 month G2019S (line 340) and R1441C (line 574) LRRK2 transgenic mice (TG) and their non-transgenic littermates (NTG). Blots were probed with a pan-LRRK2 antibody (clone c81-8/MJFF4) recognizing mouse and human LRRK2, or with β-tubulin as a protein loading control. Densitometric analysis was conducted to quantify the fold overexpression of human LRRK2 relative to endogenous mouse LRRK2. Total LRRK2 levels were normalized to β-tubulin levels and expressed as a percent of the corresponding NTG control. Bars represent the mean from *n*  =  2 mice per genotype.
(TIF)

Figure S2 Cellular localization of human LRRK2 mRNA in the ventral midbrain of G2019S LRRK2 transgenic mice. *In situ* hybridization of human or mouse LRRK2 and endogenous α-synuclein mRNAs with species-specific ^33^P-labeled oligonucleotide probes in the substantia nigra pars compacta (SNpc, A9), hippocampus and ventral tegmental area (VTA, A10) of 2-3 month G2019S LRRK2 transgenic mice (line 340). mRNA signals were revealed by development of sections in photo-emulsion and counter-staining with Cresyl violet. Notice that the mRNA signal for human LRRK2 is greater than that of endogenous LRRK2 in the SNpc, and that human LRRK2 is detected in VTA neurons whereas mouse LRRK2 is not. Scale bars: 10 μm (SNpc), 50 μm (hippocampus), 20 μm (VTA). *DG*, dentate gyrus.
(TIF)

Figure S3 Expression analysis of LRRK2 transgenic mice by *in situ* hybridization with species-specific ^33^P-labeled antisense oligonucleotide probes. ***A***, Localization of mouse LRRK2 mRNA throughout the brains of 2-3 month WT (line 249), R1441C (line 574) and G2019S (line 340) LRRK2 transgenic mice. ***B***, Expression pattern of mouse or human LRRK2 mRNA in the spleen and kidney of WT, R1441C and G2019S LRRK2 transgenic mice. Notice the endogenous expression of LRRK2 throughout the spleen and kidney. *Olf*, olfactory bulb; *Str*, striatum; *Ctx*, cerebral cortex; *Hip*, hippocampus; *Cb*, cerebellum.
(TIF)

Figure S4 Stereological analysis of dopaminergic neurons in the substantia nigra pars compacta of aged LRRK2 transgenic mice. Unbiased stereological analysis of TH+ and Nissl+ neurons in the pars compacta fails to reveal dopaminergic neuronal loss in 15-21 month WT (lines 249 and 27), R1441C (line 546) and G2019S (line 1128) LRRK2 transgenic mice (TG) compared to their age-matched non-transgenic littermates (NTG). Bars represent the mean ± SEM. Numbers of mice used per genotype: lines 249 (*n*  =  5), 27 (*n*  =  3-4), 546 (*n*  =  4-6) and 1128 (*n*  =  6). There are no statistically significant differences between TG and NTG groups.
(TIF)

Figure S5 Striatal dopaminergic nerve terminals in G2019S LRRK2 transgenic mice. TH+ immunoreactivity in the striatum of 19-20 month G2019S LRRK2 mice (TG, line 340) compared to their non-transgenic littermate mice (NTG). The optical density of TH+ immunoreactivity was quantified by densitometric analysis of every fourth section throughout the left and right striatum for each mouse using NIH ImageJ software. Bars represent the mean ± SEM (*n*  =  5-6 mice/genotype).
(TIF)

Figure S6 HPLC analysis of biogenic amines in LRRK2 transgenic mice. ***A***, Biogenic amine levels in the prefrontal cortex of 14-15 month G2019S LRRK2 mice (line 340) compared to their NTG littermates by HPLC (*n*  =  8 mice/genotype). ***B***, Biogenic amine levels in the striatum and cerebral cortex of 19-20 month R1441C LRRK2 mice (line 574) compared to their NTG littermates by HPLC (*n*  =  7-11 mice/genotype). ***C***, Levels of dopamine and its metabolites, DOPAC and HVA, in the striatum, olfactory bulb and cerebral cortex of 16-17 month WT LRRK2 mice (line 249) compared to their non-transgenic (NTG) littermates by HPLC (*n*  =  6 = 7 mice/genotype). Bars represent the mean ± SEM. *P<0.05 or **P<0.01 comparing TG with NTG for each biogenic amine as indicated.
(TIF)

Figure S7 Lack of PD-related neuropathology in aged G2019S LRRK2 transgenic mice. Sections containing the substantia nigra and cerebral cortex from 23-24 month-old G2019S LRRK2 transgenic (TG, line 340) or non-transgenic (NTG) mice were stained by immunohistochemistry with antibodies for (***A***) mouse α-synuclein, (***B***) phospho-α-synuclein (pSer129), (***C***) mouse ubiquitin, (***D***) mouse tau, (***E***) phospho-tau (PHF-1; pSer396/Ser404) and (***F***) GFAP, and by histological staining with hematoxylin (***G***). There are no distinguishable differences between NTG and TG mice. Scale bar: 100 μm.
(TIF)

Figure S8 Accumulation of autophagic vacuoles in the striatum of G2019S LRRK2 transgenic mice. Transmission electron microscopic analysis of striatal tissue from 17-18 month-old G2019S LRRK2 transgenic mice (line 340) revealing the accumulation of autophagic vacuoles (indicated by *) within (**A-B**) neuronal soma and (**C-D**) axonal-rich regions. Nuclei (*nuc*), axons (*ax*) and normal mitochondria (*m*) are indicated. (**E**) Quantitation of the density of autophagic vacuoles in equivalent regions of striatum from 17-18 month-old G2019S LRRK2 transgenic (TG) mice relative to their non-transgenic (NTG) littermates. Bars represent the mean ± SEM (*n*  =  3 mice/genotype). *P<0.01 comparing TG and NTG mice. Scale bars: 200 nm (A-D).
(TIF)

Figure S9 Autophagic vacuoles accumulate in neuronal soma and axonal processes in G2019S LRRK2 transgenic mice. Transmission electron microscopic images of cerebral cortex tissue from 17-26 month-old G2019S LRRK2 transgenic mice (line 340) highlighting the accumulation of autophagic vacuoles (indicated by *) within (**A-B**) neuronal soma and (**C-D**) axonal processes. Nuclei (*nuc*), axons (*ax*) and normal mitochondria (*m*) are indicated. Scale bars: 1 µm (A-D).
(TIF)

Figure S10 Mitochondrial abnormalities in G2019S LRRK2 transgenic mice. Transmission electron microscopic images showing representative examples of a morphologically normal mitochondrion (class I), a damaged mitochondrion (class II), or abnormal condensed mitochondrial aggregates in the cerebral cortex of 17-26 month G2019S LRRK2 transgenic mice (line 340). Scale bar: 250 nm.
(TIF)
