# Supplementary figures and images for: Dopaminergic Neuronal Loss, Reduced Neurite Complexity and Autophagic Abnormalities in Transgenic Mice Expressing G2019S Mutant LRRK2
Source: PLoS One. 2011 Apr 6;6(4):e18568. doi: 10.1371/journal.pone.0018568 (PMC3071839; doi:10.1371/journal.pone.0018568)

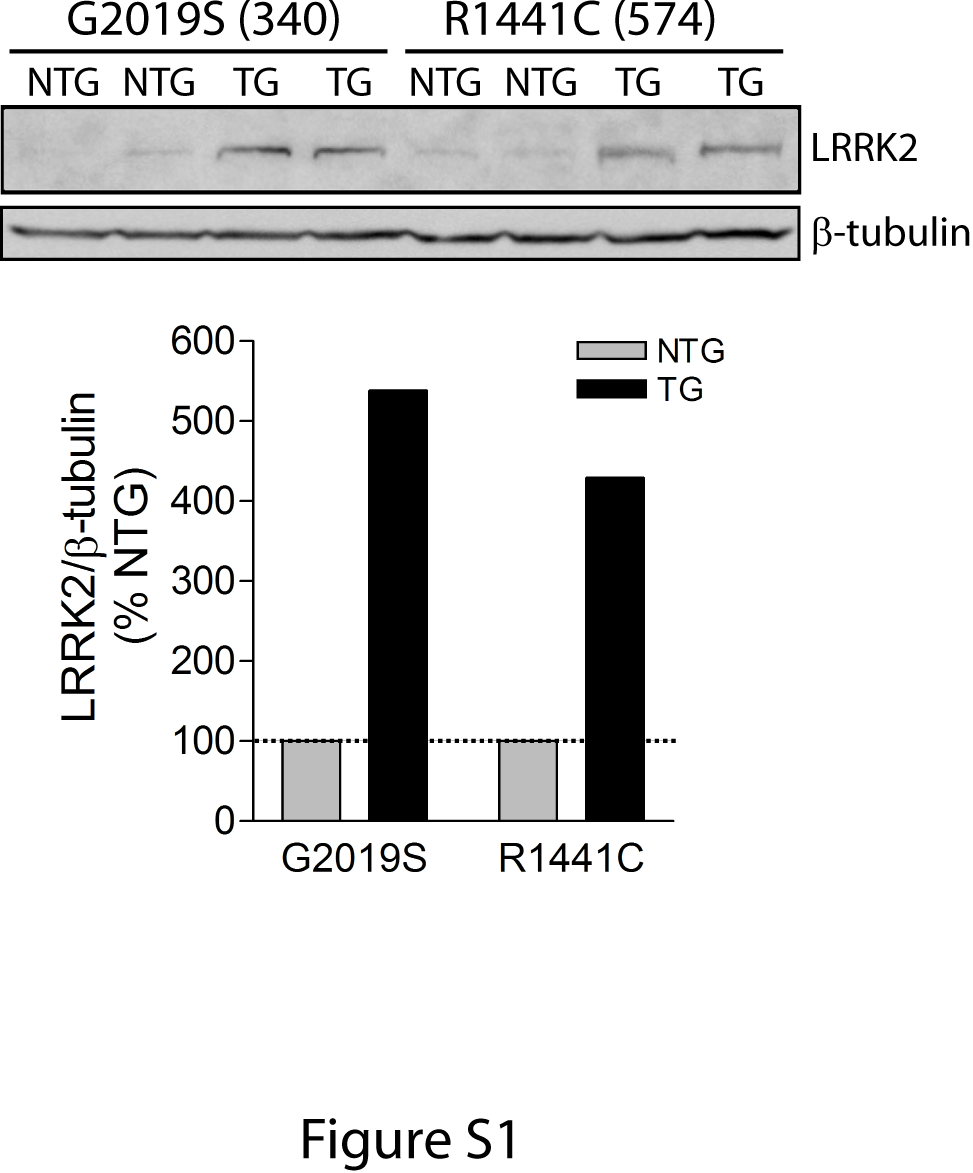

Supplement: Figure S1 — Quantitation of LRRK2 protein levels in LRRK2 transgenic mice. Western blot analysis of soluble brain extracts (75 μg protein) derived from hemi-brains of 3-4 month G2019S (line 340) and R1441C (line 574) LRRK2 transgenic mice (TG) and their non-transgenic littermates (NTG). Blots were probed with a pan-LRRK2 antibody (clone c81-8/MJFF4) recognizing mouse and human LRRK2, or with β-tubulin as a protein loading control. Densitometric analysis was conducted to quantify the fold overexpression of human LRRK2 relative to endogenous mouse LRRK2. Total LRRK2 levels were normalized to β-tubulin levels and expressed as a percent of the corresponding NTG control. Bars represent the mean from n = 2 mice per genotype. (TIF) [file pone.0018568.s001.tif]

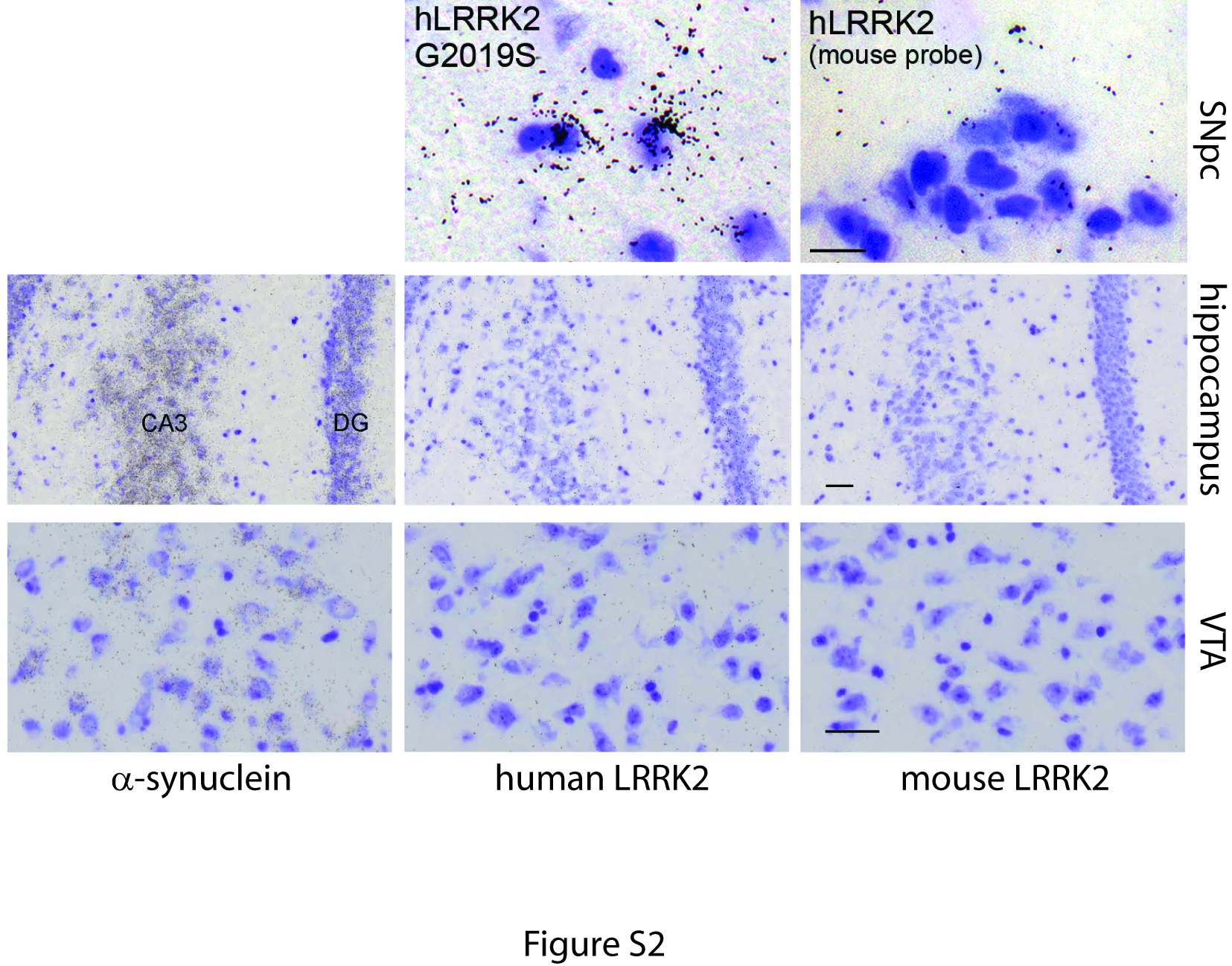

Supplement: Figure S2 — Cellular localization of human LRRK2 mRNA in the ventral midbrain of G2019S LRRK2 transgenic mice. In situ hybridization of human or mouse LRRK2 and endogenous α-synuclein mRNAs with species-specific 33P-labeled oligonucleotide probes in the substantia nigra pars compacta (SNpc, A9), hippocampus and ventral tegmental area (VTA, A10) of 2-3 month G2019S LRRK2 transgenic mice (line 340). mRNA signals were revealed by development of sections in photo-emulsion and counter-staining with Cresyl violet. Notice that the mRNA signal for human LRRK2 is greater than that of endogenous LRRK2 in the SNpc, and that human LRRK2 is detected in VTA neurons whereas mouse LRRK2 is not. Scale bars: 10 μm (SNpc), 50 μm (hippocampus), 20 μm (VTA). DG, dentate gyrus. (TIF) [file pone.0018568.s002.tif]

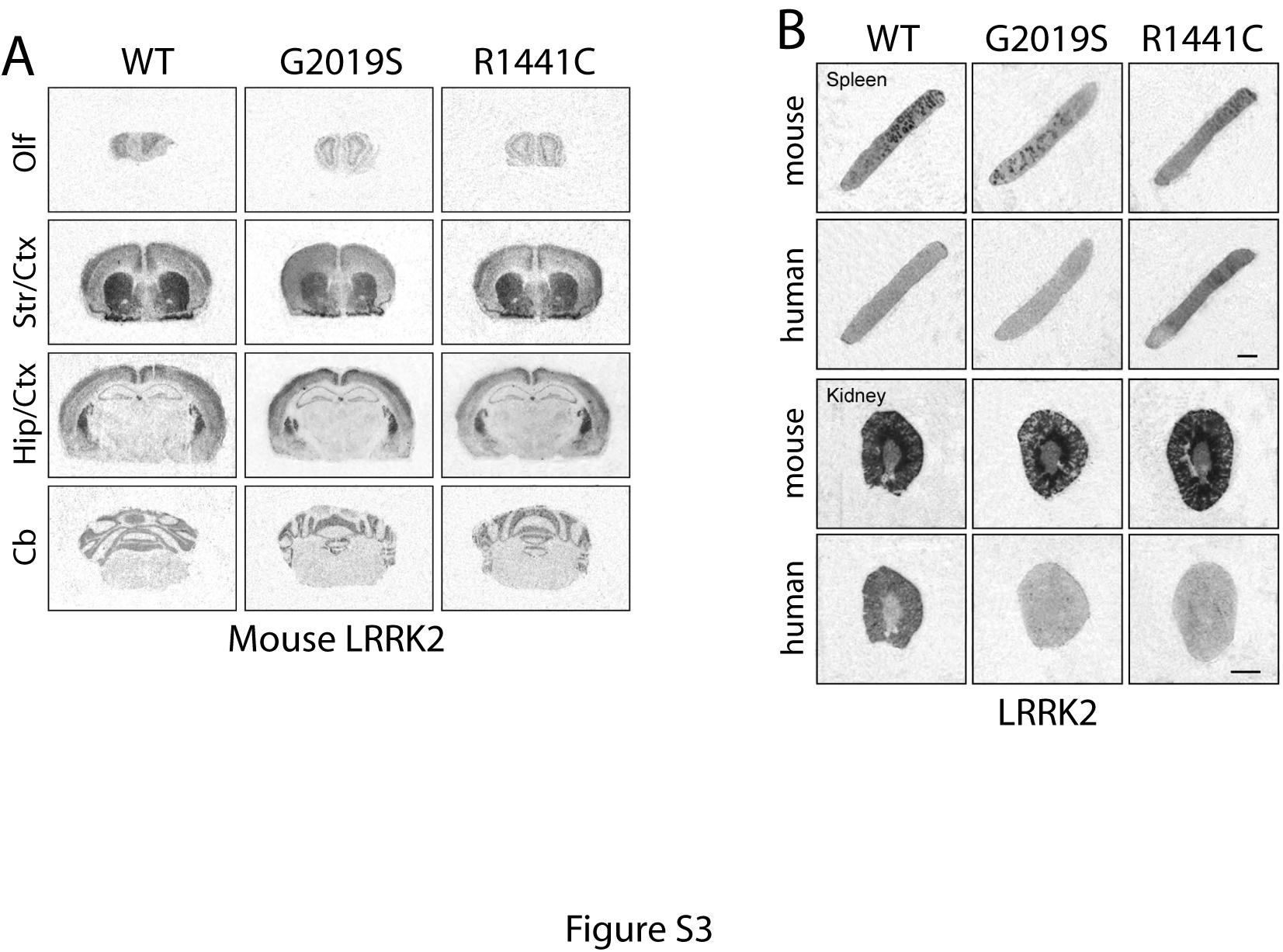

Supplement: Figure S3 — Expression analysis of LRRK2 transgenic mice by in situ hybridization with species-specific 33P-labeled antisense oligonucleotide probes. A, Localization of mouse LRRK2 mRNA throughout the brains of 2-3 month WT (line 249), R1441C (line 574) and G2019S (line 340) LRRK2 transgenic mice. B, Expression pattern of mouse or human LRRK2 mRNA in the spleen and kidney of WT, R1441C and G2019S LRRK2 transgenic mice. Notice the endogenous expression of LRRK2 throughout the spleen and kidney. Olf, olfactory bulb; Str, striatum; Ctx, cerebral cortex; Hip, hippocampus; Cb, cerebellum. (TIF) [file pone.0018568.s003.tif]

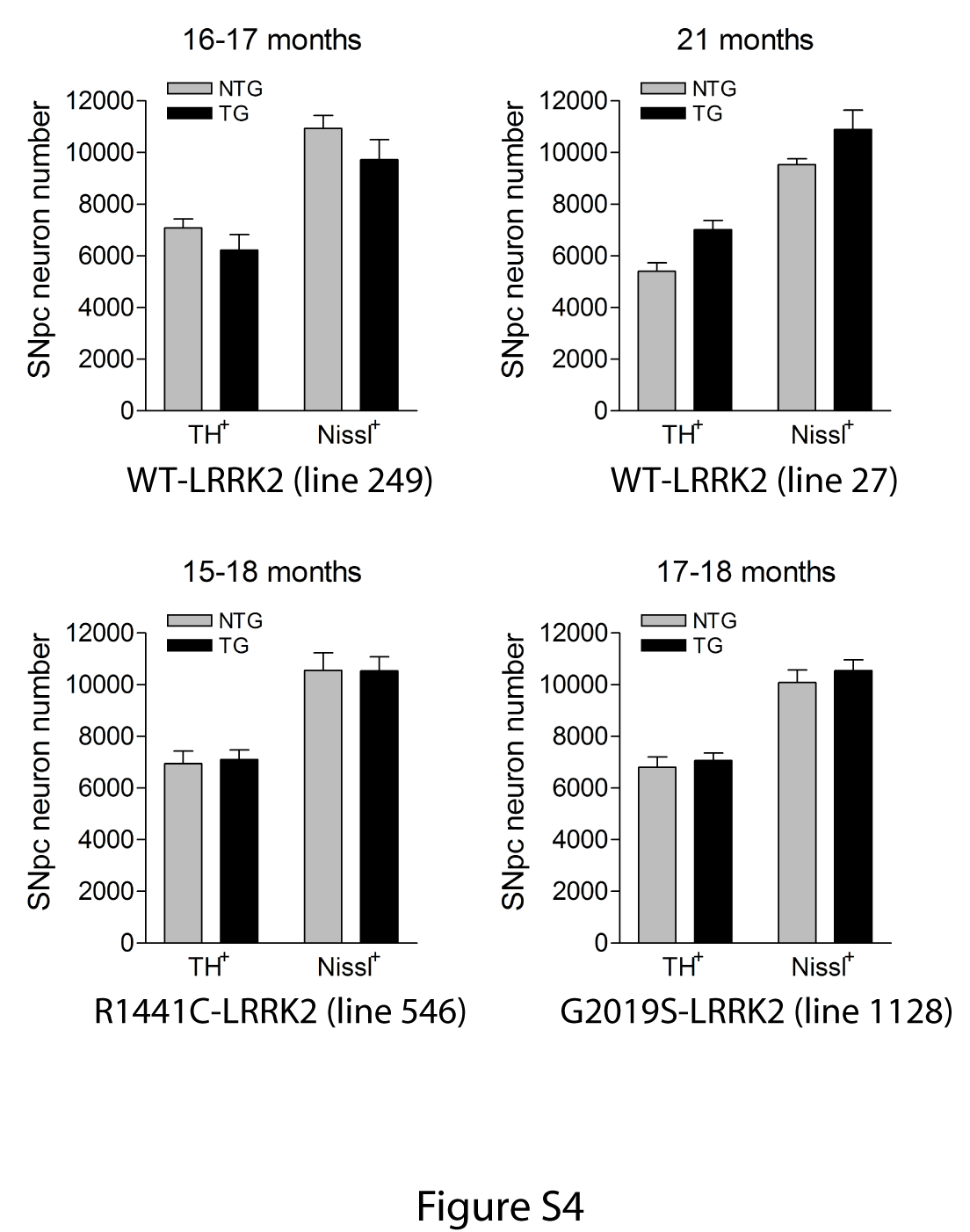

Supplement: Figure S4 — Stereological analysis of dopaminergic neurons in the substantia nigra pars compacta of aged LRRK2 transgenic mice. Unbiased stereological analysis of TH+ and Nissl+ neurons in the pars compacta fails to reveal dopaminergic neuronal loss in 15-21 month WT (lines 249 and 27), R1441C (line 546) and G2019S (line 1128) LRRK2 transgenic mice (TG) compared to their age-matched non-transgenic littermates (NTG). Bars represent the mean ± SEM. Numbers of mice used per genotype: lines 249 (n = 5), 27 (n = 3-4), 546 (n = 4-6) and 1128 (n = 6). There are no statistically significant differences between TG and NTG groups. (TIF) [file pone.0018568.s004.tif]

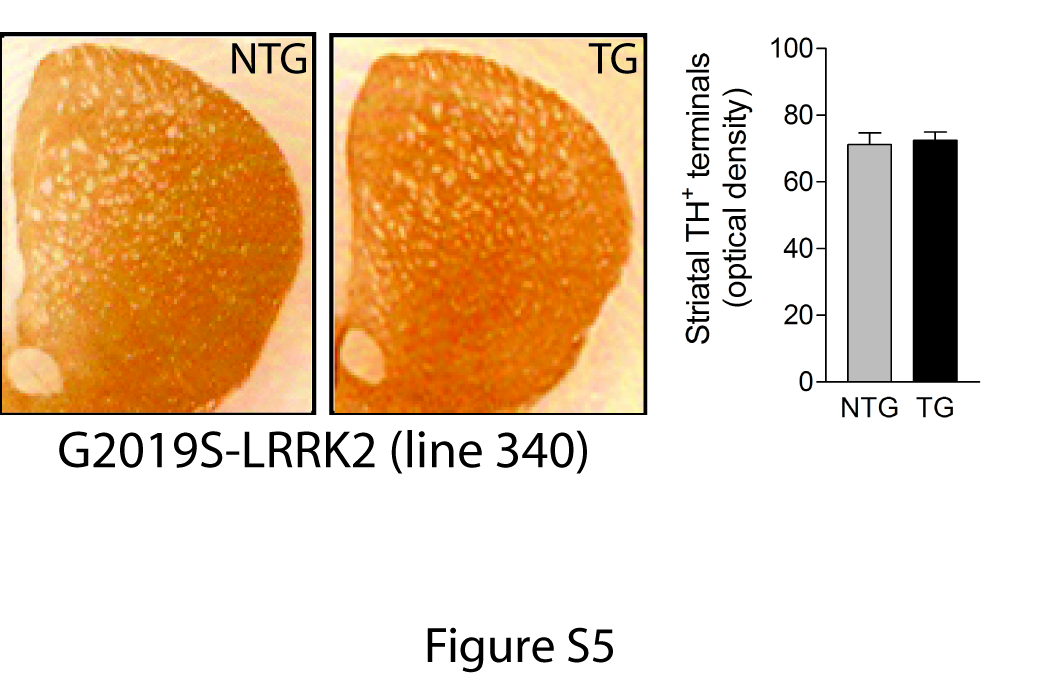

Supplement: Figure S5 — Striatal dopaminergic nerve terminals in G2019S LRRK2 transgenic mice. TH+ immunoreactivity in the striatum of 19-20 month G2019S LRRK2 mice (TG, line 340) compared to their non-transgenic littermate mice (NTG). The optical density of TH+ immunoreactivity was quantified by densitometric analysis of every fourth section throughout the left and right striatum for each mouse using NIH ImageJ software. Bars represent the mean ± SEM (n = 5-6 mice/genotype). (TIF) [file pone.0018568.s005.tif]

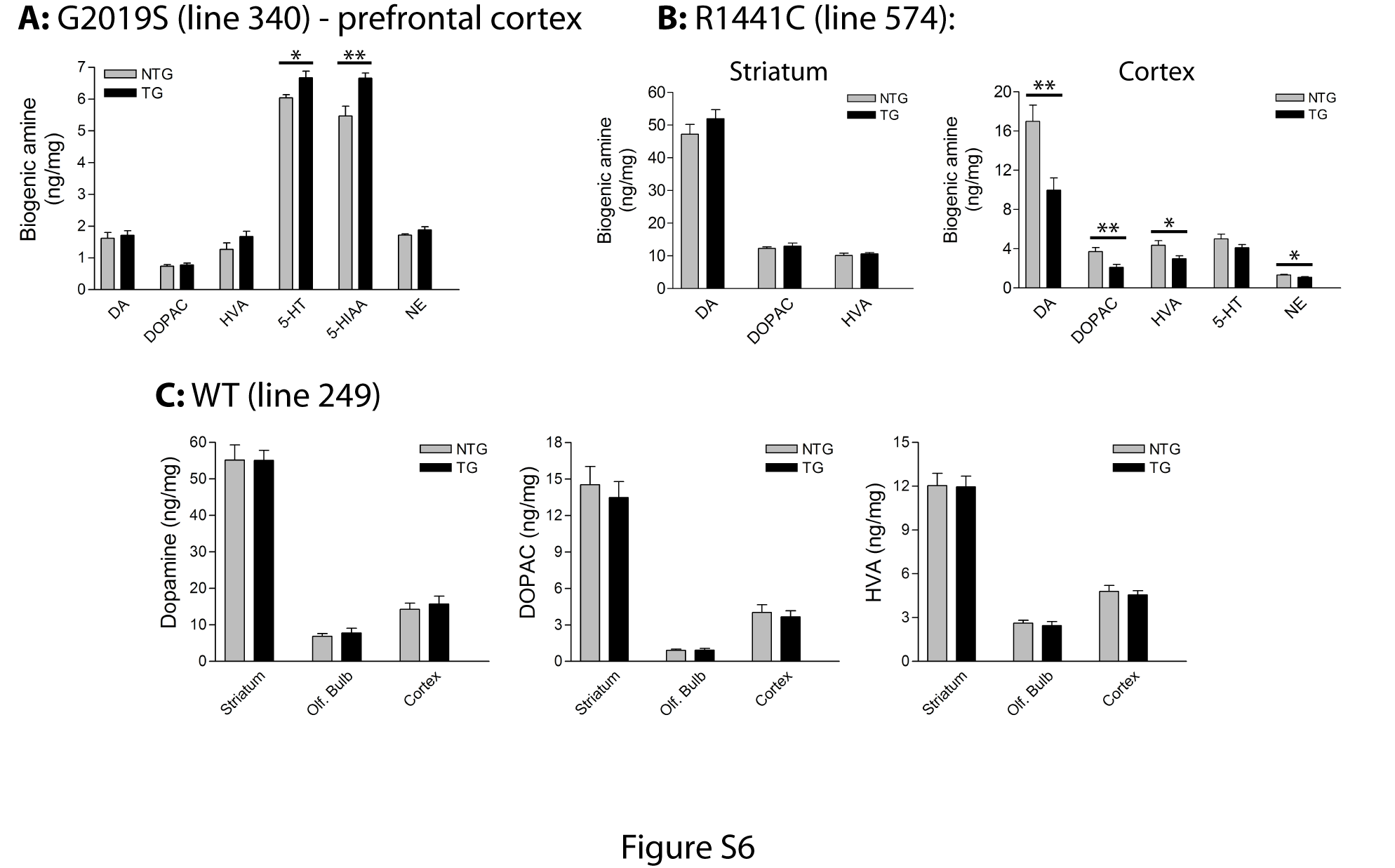

Supplement: Figure S6 — HPLC analysis of biogenic amines in LRRK2 transgenic mice. A, Biogenic amine levels in the prefrontal cortex of 14-15 month G2019S LRRK2 mice (line 340) compared to their NTG littermates by HPLC (n = 8 mice/genotype). B, Biogenic amine levels in the striatum and cerebral cortex of 19-20 month R1441C LRRK2 mice (line 574) compared to their NTG littermates by HPLC (n = 7-11 mice/genotype). C, Levels of dopamine and its metabolites, DOPAC and HVA, in the striatum, olfactory bulb and cerebral cortex of 16-17 month WT LRRK2 mice (line 249) compared to their non-transgenic (NTG) littermates by HPLC (n = 6 = 7 mice/genotype). Bars represent the mean ± SEM. *P<0.05 or **P<0.01 comparing TG with NTG for each biogenic amine as indicated. (TIF) [file pone.0018568.s006.tif]

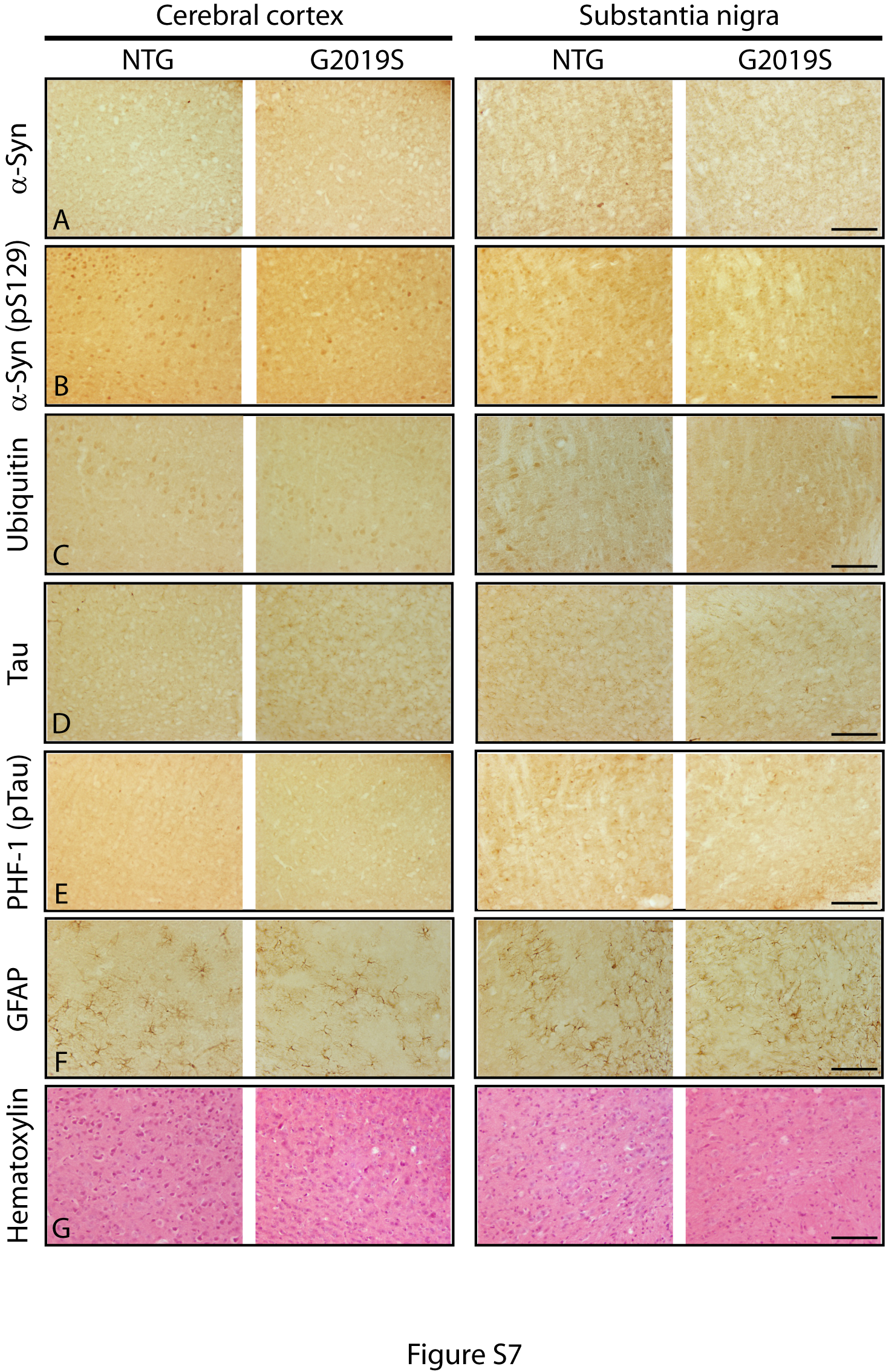

Supplement: Figure S7 — Lack of PD-related neuropathology in aged G2019S LRRK2 transgenic mice. Sections containing the substantia nigra and cerebral cortex from 23-24 month-old G2019S LRRK2 transgenic (TG, line 340) or non-transgenic (NTG) mice were stained by immunohistochemistry with antibodies for (A) mouse α-synuclein, (B) phospho-α-synuclein (pSer129), (C) mouse ubiquitin, (D) mouse tau, (E) phospho-tau (PHF-1; pSer396/Ser404) and (F) GFAP, and by histological staining with hematoxylin (G). There are no distinguishable differences between NTG and TG mice. Scale bar: 100 μm. (TIF) [file pone.0018568.s007.tif]

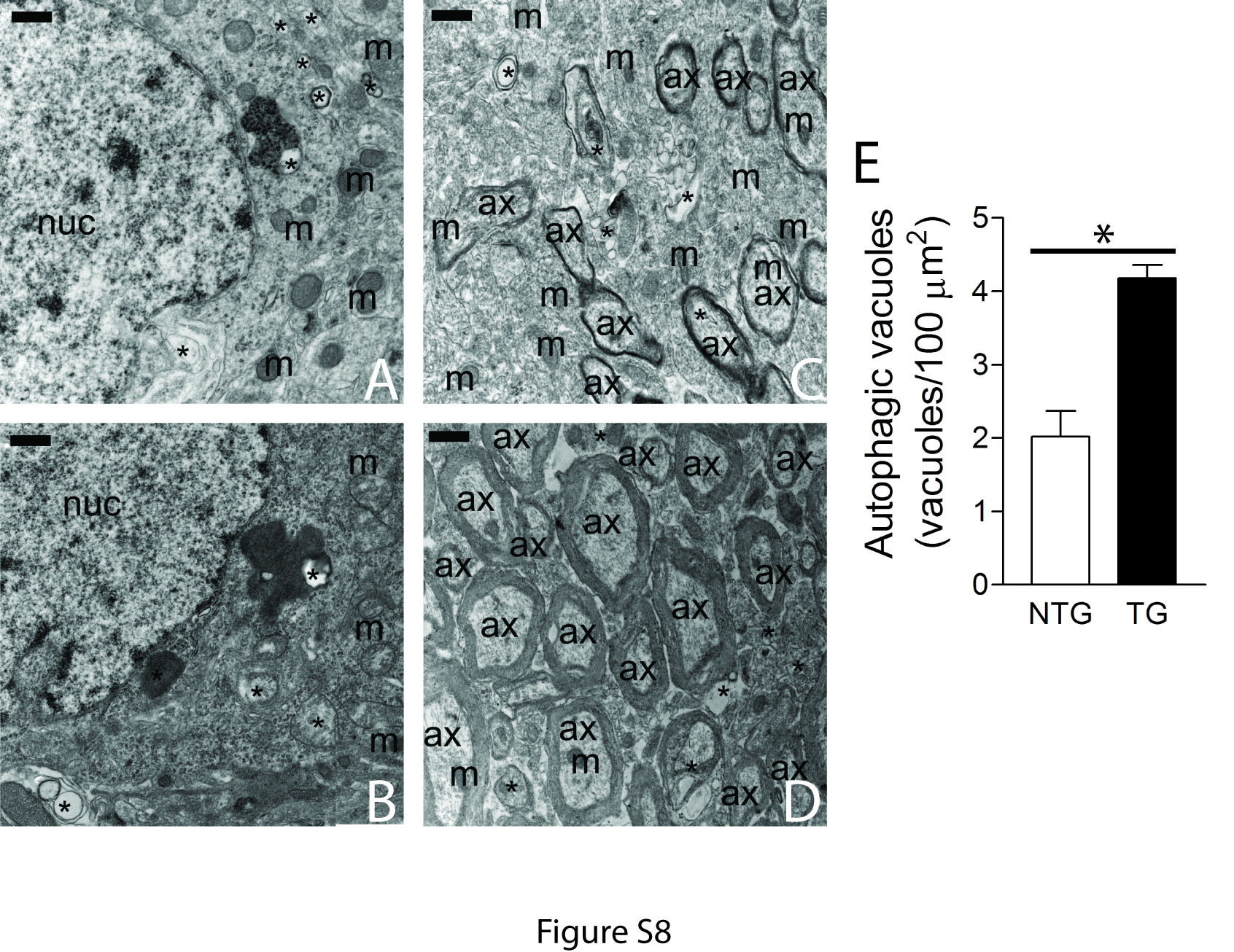

Supplement: Figure S8 — Accumulation of autophagic vacuoles in the striatum of G2019S LRRK2 transgenic mice. Transmission electron microscopic analysis of striatal tissue from 17-18 month-old G2019S LRRK2 transgenic mice (line 340) revealing the accumulation of autophagic vacuoles (indicated by *) within (A-B) neuronal soma and (C-D) axonal-rich regions. Nuclei (nuc), axons (ax) and normal mitochondria (m) are indicated. (E) Quantitation of the density of autophagic vacuoles in equivalent regions of striatum from 17-18 month-old G2019S LRRK2 transgenic (TG) mice relative to their non-transgenic (NTG) littermates. Bars represent the mean ± SEM (n = 3 mice/genotype). *P<0.01 comparing TG and NTG mice. Scale bars: 200 nm (A-D). (TIF) [file pone.0018568.s008.tif]

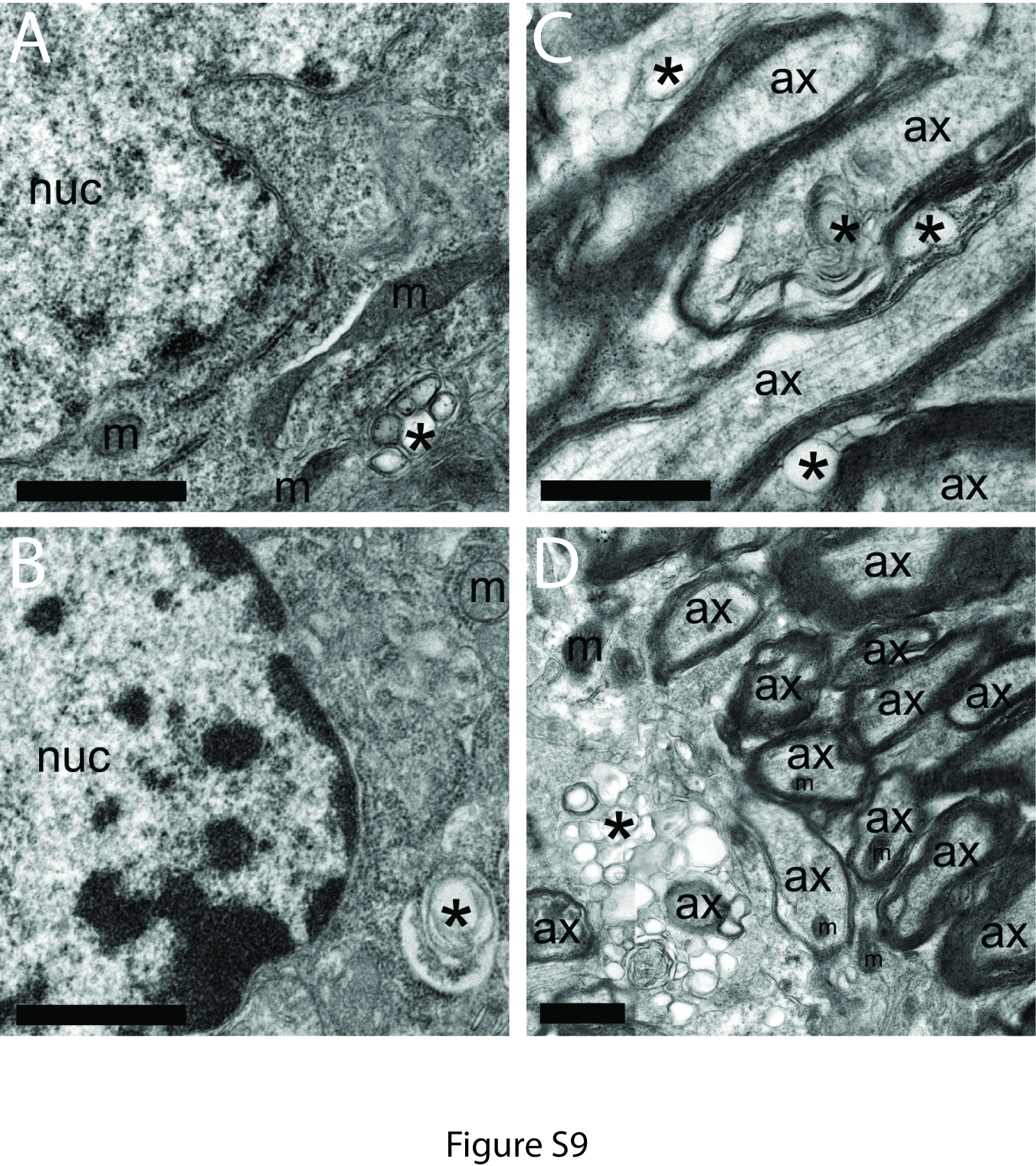

Supplement: Figure S9 — Autophagic vacuoles accumulate in neuronal soma and axonal processes in G2019S LRRK2 transgenic mice. Transmission electron microscopic images of cerebral cortex tissue from 17-26 month-old G2019S LRRK2 transgenic mice (line 340) highlighting the accumulation of autophagic vacuoles (indicated by *) within (A-B) neuronal soma and (C-D) axonal processes. Nuclei (nuc), axons (ax) and normal mitochondria (m) are indicated. Scale bars: 1 µm (A-D). (TIF) [file pone.0018568.s009.tif]

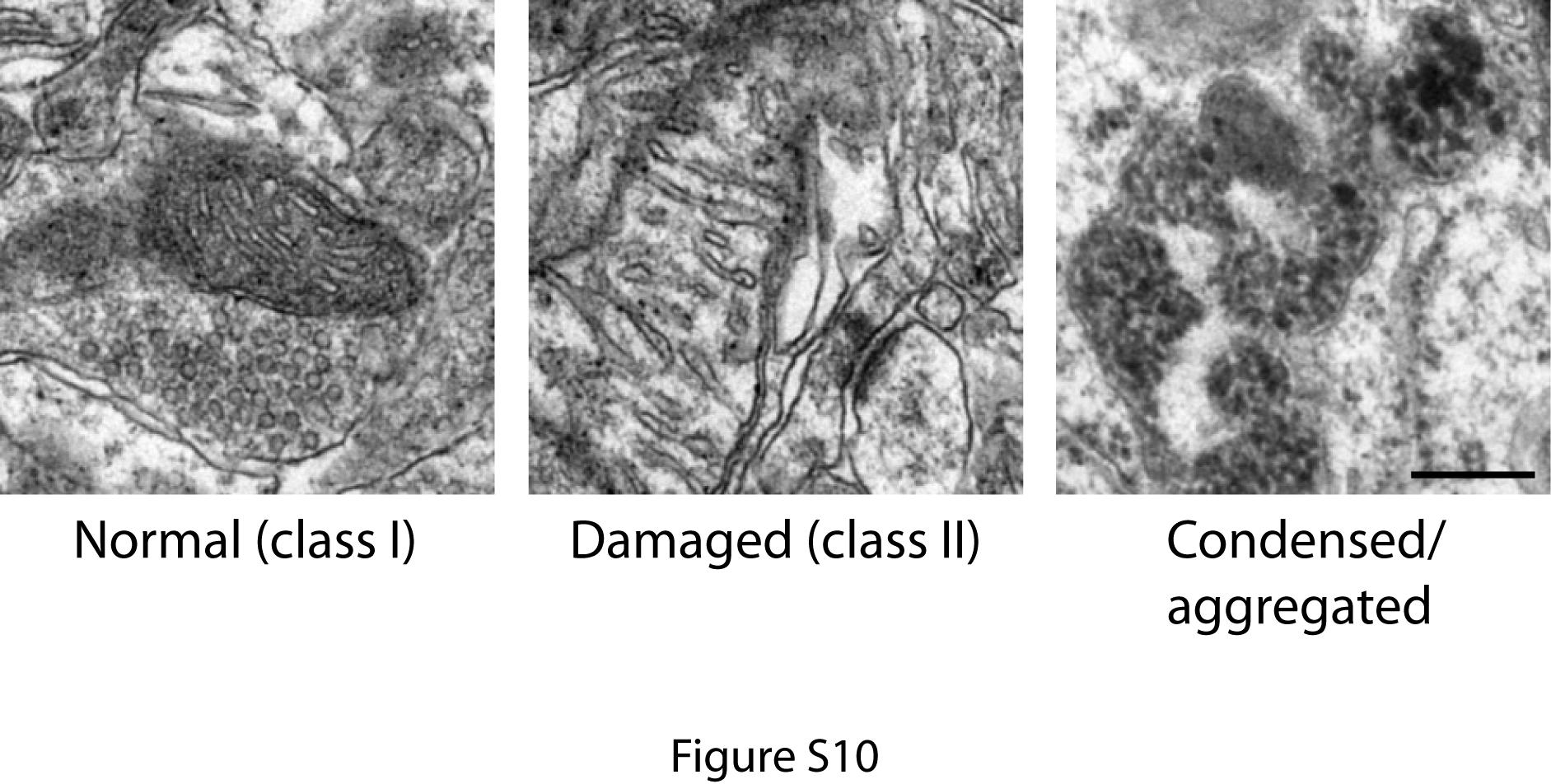

Supplement: Figure S10 — Mitochondrial abnormalities in G2019S LRRK2 transgenic mice. Transmission electron microscopic images showing representative examples of a morphologically normal mitochondrion (class I), a damaged mitochondrion (class II), or abnormal condensed mitochondrial aggregates in the cerebral cortex of 17-26 month G2019S LRRK2 transgenic mice (line 340). Scale bar: 250 nm. (TIF) [file pone.0018568.s010.tif]
